# Strong Membrane Permeabilization Activity Can Reduce Selectivity of Cyclic Antimicrobial Peptides

**DOI:** 10.1021/acs.jpcb.4c05019

**Published:** 2025-02-19

**Authors:** Katharina Beck, Janina Nandy, Maria Hoernke

**Affiliations:** † Pharmaceutical Technology and Biopharmacy, Institute of Pharmaceutical Sciences, University of Freiburg, 79104 Freiburg im Breisgau, Germany; ‡ Physiology, Institute of Theoretical Medicine, University of Augsburg, 86159 Augsburg, Germany; § Experimental Physics I, Institute of Physics, University of Augsburg, 86159 Augsburg, Germany; ⊥ Physical Chemistry, Martin-Luther-Universität, 06120 Halle (S.), Germany

## Abstract

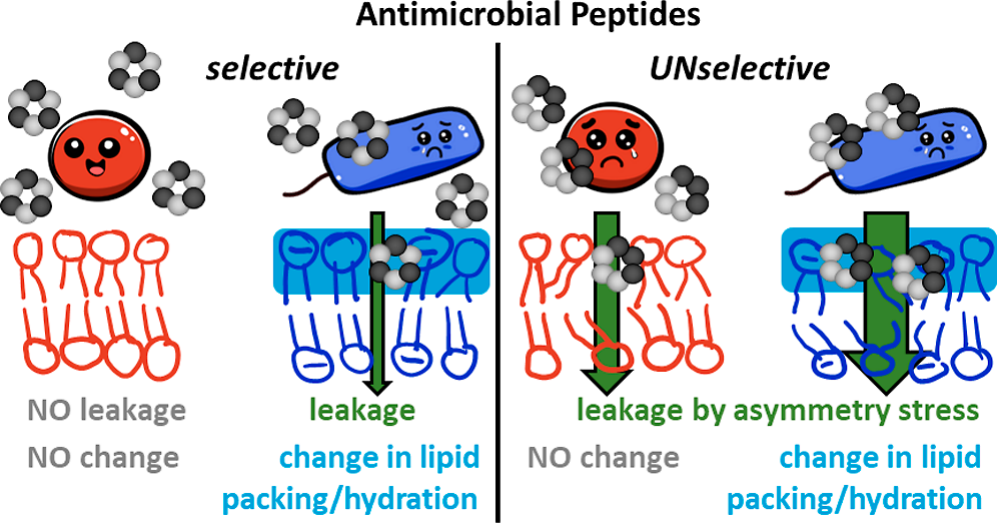

Selectivity is a key requirement for membrane-active antimicrobials to be viable in therapeutic contexts. Therefore, the rational design or suitable selection of new compounds requires adequate mechanistic understanding of peptide selectivity. In this study, we compare two similar cyclic peptides that differ only in the arrangement of their three hydrophobic tryptophan (W) and three positively charged arginine (R) residues, yet exhibit different selectivities. This family of peptides has previously been shown to target the cytoplasmic membrane of bacteria, but not to act directly by membrane permeabilization. We have systematically studied and compared the interactions of the two peptides with zwitterionic phosphatidylcholine (PC) and negatively charged phosphatidylglycerol/phosphatidylethanolamine (PG/PE) model membranes using various biophysical methods to elucidate the mechanism of the selectivity. Like many antimicrobial peptides, the cyclic, cationic hexapeptides investigated here bind more efficiently to negatively charged membranes than to zwitterionic ones. Consequently, the two peptides induce vesicle leakage, changes in lipid packing, vesicle aggregation, and vesicle fusion predominantly in binary, negatively charged PG/PE membranes. The peptide with the larger hydrophobic molecular surface (three adjacent W residues) causes all these investigated effects more efficiently. In particular, it induces leakage by asymmetry stress and/or leaky fusion in zwitterionic and charged membranes, which may contribute to high activity but reduces selectivity. The unselective type of leakage appears to be driven by the more pronounced insertion into the lipid layer, facilitated by the larger hydrophobic surface of the peptide. Therefore, avoiding local accumulation of hydrophobic residues might improve the selectivity of future membrane-active compounds.

## 1 Introduction

The growing resistance of pathogens to conventional antibiotics threatens adequate patient care worldwide.^1^ This highlights the importance of advancing research and development efforts toward new active compounds that do not cause resistance. Among these, antimicrobial peptides (AMPs) have emerged as a promising class of compounds for which the development of resistance is rarely observed.^2−6^ The typically small, amphipathic peptides are commonly thought to mainly target the microbial cell membrane. However, not only antimicrobial activity but also selectivity is important in the search for novel antibiotics.^7−9^ Treatment of infectious diseases without side effects is only possible by combining both aspects. How AMPs obtain their selectivity is often unexplored and unknown.

Recent advances in employing genetic material or enzymes for vaccines and therapeutic treatments require efficient transfection.^10^ One strategy is to use cell-penetrating peptides (CPPs) to transport cargo across cytoplasmic or endosomal membranes. Again, for the potential application of CPPs, their activity and toxicity, *i.e.* selectivity, need to be finely tuned and well understood.^11,12^

The peptides in our work differ from cyclic lipopeptides, such as daptomycin, which is an approved reserve antibiotic, by their hydrophobic anchors.^13^ Also the recently reported cyclic peptide lugdunin exhibits a mechanisms that relies on its unusual potential to stack and form tubes.^14,15^ This study focuses on two candidates from the class of cyclic R- and W-rich peptides. Linear and cyclic hexapeptides rich in arginine (R) and tryptophan (W) have been widely studied and are considered promising candidates as antimicrobials.^11,16,17^ Natural and designed sequences of R and W can also yield efficient CPPs.^11,18−20^ While for antimicrobials, a certain membrane permeabilization is seen as advantageous, CPPs are thought to internalize into cells without leakage. R- and W-rich peptides can either be antimicrobial, cell penetrating, or both. Many are also cytotoxic and hemolytic (reviewed in^11,16^). For both antimicrobial and cell-penetrating peptides, enhanced activity often correlates with increased toxicity, *i.e.* reduced selectivity,^8,11,21−25^ illustrating the toxicity-efficiency dilemma also discussed for biomimetic transfection polymers.^26−28^ The amphipathic structure of R- and W-rich peptides, consisting of aromatic and charged residues, enables them to interact with membranes. Among these cyclic hexapeptides, c-RRRWFW (cWFW) was identified as the most antimicrobially active peptide^29^ after Dathe *et al.* observed an improvement of the bacterial activity and bacterial selectivity upon cyclization of these sequences.^30−32^ Additionally, cyclic peptides are also less susceptible to enzymatic degradation. The cyclic hexapeptides lead to a rapid killing of Gram-positive and Gram-negative bacteria, showing a bactericidal effect at the minimal inhibitory concentration (MIC).^33,34^ They efficiently translocate the outer membrane of *Escherichia coli* and target in particular the cytoplasmic membrane.^33,35,36^ Scheinpflug *et al.* observed that cWFW strongly partitions into PE/cardiolipin-rich membrane regions and reduces membrane fluidity, *i.e.* changes laurdan fluorescence, in cells.^36,37^ Scheinpflug *et al.* also found the dissociation of essential membrane proteins from the cytoplasmic membrane, the inhibition of cell wall synthesis, and autolysis.^36,37^ Similar behavior was found for the related linear RWRWRW peptide (also called M196).^38^ In model membranes, the peptides interact presumably by electrostatic attraction between cationic residues and negatively charged lipid. However, their insertion is also driven by the hydrophobic effect. In PG/PE model membranes, the peptides enhance lateral lipid demixing after binding and the formation of PG-rich clusters.^39−41^ Generally, antimicrobial peptides and their analogues are believed to affect the physicochemical properties and function of membranes, instead of binding and interfering with well-defined molecular structures. It remains unclear, why mutations and changes in the sequence of AMPs and CPPs can still render them inactive or unselective.

This paper addresses why certain R- and W-rich peptides exhibit high selectivity, while others, despite their favorable antimicrobial properties, lack of clinical relevance. The aim of our work is to expand our understanding why similar peptides can display different selectivity, thereby increasing our knowledge of the mechanism of action and selectivity of membrane-active antimicrobials and cell-penetrating peptides in general.

We focus on two structurally related antimicrobial, cyclic hexapeptides: c-RRRWWW (cR_3_W_3_) and c-RWRWRW (c(RW)_3_). Their linear variants are efficient antimicrobials (RRRWWW) or transfectants (RWRWRW).^30^ For the alternating sequence, three repeats seem an optimum between activity and toxicity.^42−44^

Despite their minor differences, not in composition but in the arrangement of amino acids, the two peptides differ in antimicrobial activity and selectivity. Junkers *et al.* studied the activity of the peptides against bacterial and eukaryotic cells and found a minimal inhibitory concentration (MIC) against *E. coli* of 11 μM for cR_3_W_3_ and 23 μM for c(RW)_3_ respectively. They also determined the toxicity of the two peptides on HeLa cells to assess potential side effects, and found a decrease in cell viability of 14% for cR_3_W_3_ and 1% for c(RW)_3_.^33^ In summary, cR_3_W_3_ has high antimicrobial activity, while c(RW)_3_ has notable selectivity.

It is thought that the peptides accumulate on the negatively charged bacterial membrane of the bacteria due to their cationic charge, and their amphipathic nature allows them to insert into the lipid membrane.^33^ In an NMR study, the charged residues and the polar backbone of cWFW were located in the headgroup region with the aromatic side chains adjacent to the hydrophobic compartment.^45^ Our hypothesis is that the minor sequence variations between cR_3_W_3_ and c(RW)_3_ are responsible for their different interactions with membranes and the resulting selectivity. Reverse phase retention times revealed the hydrophobicity and amphipathicity of the peptides. The interaction with the hydrophobic phase was reduced for c(RW)_3_ compared to cR_3_W_3_.^33^ The adjacent tryptophan residues of cR_3_W_3_ form an aromatic cluster, resulting in a larger hydrophobic surface of the peptide. Junkes *et al.* were already able to demonstrate a deeper insertion into the lipid bilayer for cR_3_W_3_ compared to c(RW)_3_ based on the tryptophan fluorescence.^33^

We have previously characterized the permeabilization induced by cR_3_W_3_ in negatively charged model membranes. We mainly found membrane permeabilization due to leaky fusion in POPG/POPE vesicles and another slower leakage effect in POPG/POPC vesicles.^46^

Here, we will first characterize the adsorption and insertion of both peptides to lipid monolayers and compare the total binding to lipid bilayers. We will discuss different binding and the resulting vesicle fusion and aggregation. Subsequently, we will use laurdan fluorescence spectroscopy to describe changes in lipid headgroup packing and hydration in response to peptide binding. Finally, a calcein leakage assay is used to comprehensively review the permeabilization induced by cR_3_W_3_ and c(RW)_3_ in model membranes. We will discuss the mechanisms of action with particular attention to the peptides selectivity. For this purpose, we use vesicles composed of zwitterionic PC or PE and negatively charged PG; characteristic lipids that are abundant in mammalian or bacterial membranes, respectively.^47,48^ This approach aims to elucidate potential selectivity mechanisms.

Upon establishing these findings, we will discuss: (I) different membrane binding and secondary effects as a function of lipid composition, (II) differences between the peptides in their leakage mechanism. En route, the mechanism of antibacterial activity previously investigated in bacteria will be confirmed by our more detailed model studies. (III) We finally propose a balance of leakage and other effects that results in the differing selectivities.

## 2 Materials and Methods

### 2.1 Materials

1-Palmitoyl-2-oleoyl-*sn*-glycero-3-phospho-(1′-rac-glycerol) sodium salt (POPG) and 1-palmitoyl-2-oleoyl-*sn*-glycero-3-phosphoethanolamine (POPE) were purchased as a chloroform solution from Avanti Polar Lipids (Alabaster, AL, USA). 1-Palmitoyl-2-oleoyl-*sn*-glycero-3-phosphocholine (POPC), 1,2-dimyristoyl-*sn*-glycero-3-phosphocholine (DMPC), 1,2-dimyristoyl-*sn*-glycero-3-phosphoethanolamine (DMPE), and 1,2-dimyristoyl-*sn*-glycero-3-phospho-(1′-rac-glycerol) sodium salt (DMPG) were provided as lyophilized powder from Lipoid GmbH (Ludwigshafen, Germany). 1,2-Dihexadecanoyl-*sn*-glycero-3-phosphoethanolamine triethylammonium salt (Rhodamine DHPE) and *N*-(7-nitrobenz-2-oxa-1,3-diazol-4-yl)-1,2-dihexadecanoyl-*sn*-glycero-3-phosphoethanolamine triethylammonium salt (NBD-PE) were purchased from Thermo Fisher Scientific Inc. (Waltham, MA, USA). Laurdan, calcein, and ethylenediaminetetraacetic acid (EDTA) were purchased from Sigma-Aldrich (St. Louis, MO, USA). Chloroform (HPCL grade), 2-amino-2-(hydroxymethyl)propane-1,3-diol (TRIS), sodium chloride (NaCl), and Triton X-100 were purchased from Carl Roth GmbH (Karlsruhe, Germany). Lipids, fluorophores, and chemicals were used without further purification.

All experiments were carried out in standard TRIS buffer (10 mM TRIS, 110 mM NaCl, 0.5 mM EDTA, pH 7.4) with ultrapure water (Merck Millipore, Darmstadt, Germany).

The synthetic cyclic peptides c-RRRWWW (cR_3_W_3_) and c-RWRWRW (c(RW)_3_) were custom-synthesized by GeneCust (Boynes, France). HPLC by GeneCust confirms purity of ≥98%. Aqueous 1 mM stock solutions of the peptides were prepared and stored at −20 °C. Immediately before an experiment, they were gently thawed and further diluted with TRIS buffer.

### 2.2 Monolayer Adsorption Experiments

The adsorption of the antimicrobial peptides to model membranes was estimated by lipid monolayer experiments. Four measurements were conducted in parallel using the DeltaPi-4x tensiometer (Kibron Inc., Helsinki, Finland) with wire probes (DyneProbes, Kibron, Helsinki, Finland) in small troughs (diameter: 230 mm, height: 3 mm). The ground plate was thermostated at 20 °C. The entire experimental setup was covered by a plastic hood to avoid fluctuations in temperature and humidity. Before each measurement, the troughs were cleaned with an aqueous Hellmanex solution (Carl Roth GmbH, Karlsruhe, Germany) and thoroughly rinsed with ultrapure water. The wire probes were cleaned by briefly flaming them with a hand-held butane torch (Leifheit AG, Nassau, Germany).

To start an experiment, the troughs were filled with 1240 μL TRIS buffer and the baseline was equilibrated for at least 20 min. A freshly prepared 1 mM cloroform solution of DMPC or DMPG/DMPE (1:1) was spread on top of the aqueous subphase using a 10 μL glass syringe (Hamilton Company, Reno, NV, USA) until a desired initial surface pressure π_0_ between 5 and 60 mN/m was reached. Before proceeding, it was ensured that the lipid monolayer had a constant surface pressure for at least 30 min. A small volume of peptide stock solution was carefully added through a side injection port to achieve a final peptide concentration of 900 nM in the subphase. The surface pressure was recorded continuously up to 2 h using the DeltaGraph software (Kibron Inc., Helsinki, Finland). The surface pressure π_end_ determined approximately 35 min after peptide addition was used for the subsequent interpretation.

To quantify the peptide adsorption on the lipid monolayer, the change in surface pressure Δπ was calculated.
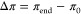
1

### 2.3 Liposome Preparation

POPC liposomes and POPG/POPE (1:1) liposomes were prepared as described previously.^46^ In certain experiments, liposomes containing additional fluorescent dyes were required.

For the preparation of these liposomes, the required lipids were first dissolved in chloroform and mixed in the appropriate ratio. If necessary, fluorescent dyes were also dissolved in chloroform and added to the lipid solution. Several glass vials were then filled with the lipid mixtures and the chloroform was removed by a rotary vacuum concentrator (RVC 2−18 CDplus, Martin Christ GmbH, Osterode am Harz, Germany) at 36 °C and additionally dried overnight under vacuum to obtain thin lipid films. Dry lipid films that were not used immediately were stored at −20 °C.

Large unilamellar vesicles (LUVs) were produced by extrusion. First, a lipid film was rehydrated with TRIS buffer through vortexing at room temperature. Unless otherwise stated, a lipid stock concentration of 10 mM was utilized. This was followed by five freeze–thaw cycles before the liposome suspension was extruded 51 times through a 80 nm polycarbonate membrane (Nuclepore Track-Etched Membranes, Whatman International Ltd., Maidstone, United Kingdom) using a LiposoFast hand extruder (Avestin, Ottawa, Canada) at room temperature.

After the preparation, the lipid concentration of the liposome suspension was assessed by the Bartlett assay.^49^ Furthermore, DLS measurements confirmed the particle size of the liposome suspension to be 110 ± 10 nm with a size distribution characterized by PDI < 0.1.

### 2.4 Dynamic Light Scattering (DLS)

A Zetasizer Nano ZS (Malvern Panalytical Ltd., Worcestershire, United Kingdom) was used to measure the hydrodynamic diameter (*Z*-average) and the polydispersity index (PDI) to asses the particle size and size distribution of liposome suspensions. The diluted liposome suspension was illuminated with a 633 nm helium−neon laser and the scattered light signal was collected at a back scattering angle of 173°. The measurements were carried out at 25 °C and the calculation, taking into account the refractive index and viscosity of the buffer, was performed directly by the instrument software.

For a DLS measurement, the liposome suspensions had to be diluted with TRIS buffer. This was done directly in the disposable cuvette (Semimicro PMMA cuvette, Brand GmbH & Co. KG, Wertheim, Germany) used for the measurement. To verify the particle size of the liposome suspension after the preparation, 5 μL of the stock solution were diluted with 1 mL TRIS buffer. Three measurements were automatically averaged by the instrument.

In order to observe the influence of the antimicrobial peptides on model membranes under the same conditions as in our other experiments, the same concentrations and incubation times were applied and analyzed. The caption of Figure 4 summarizes the concentrations in detail. Several disposable polystyrene cuvettes (Sarstedt AG & Co., KG, Nümbrecht, Germany) containing 30 μM liposome suspension were incubated with increasing concentrations of the peptides up to 24 h on a rocking shaker (Single TEC Control Shaker, INHECO, Martinsried, Germany) with 400 rpm at 25 °C. Subsequently, the particle size and size distribution of the suspension was determined.

### 2.5 Isothermal Titration Calorimetry (ITC)

To assess the total binding of the antimicrobial peptides to model membranes, isothermal titration calorimetry (ITC) measurements were performed on a VP-ITC MicroCalorimeter (Malvern Panalytical Ltd., Worcestershire, United Kingdom).

First, the stock solutions of the peptides and freshly prepared liposome suspensions were individually diluted with TRIS buffer to achieve the desired concentrations. Initial concentrations ranged from 5 to 20 mM for liposome suspensions and 0.02 to 0.1 mM for peptide solutions. The caption of Figure 2 summarizes the concentrations in detail. The samples then were thermostated at 25 °C and degassed for 4 min using the ThermoVac accessory device (Malvern Panalytical Ltd., Worcestershire, United Kingdom). The respective peptide solution was filled into the reaction cell and the liposome suspension was loaded into the syringe. Every 10 min, aliquots of 10 μL were injected into the cell with a duration of 20 s, stirred at 286 rpm, and maintained at 25.0 °C.

MicroCal Origin Analysis Software (MicroCal, Northhampton, MA, USA) was used to visualize the raw thermograms, integrate them, and calculate thermodynamic parameters. Heat of dilution was negligible and not taken into account. We employed a one-set-of-sites fitting model to provide a quantitative estimate of peptide binding to lipid membranes. This model simplifies the analysis by combining various contributions into a single apparent binding constant *K*, enthalpy Δ*H*, and stoichiometry *n*, allowing us to focus on overall trends rather than distinguishing individual contributions, such as electrostatic and hydrophobic interactions. Details are given in the Supporting Information.

### 2.6 Laurdan Fluorescence Spectroscopy

Lipid packing, hydration, and the fluidity of a lipid membrane can be assessed using laurdan fluorescence spectroscopy.^50−52^ We examined the influence of the antimicrobial peptides on these membrane properties.

The fluorescence spectra of membrane-embedded laurdan were measured using the lipid state observer (LISO). This customized spectrometer precisely controls the temperature, excites a sample at 360 nm with an ultraviolet LED, and measures the emitted light from 390 to 800 nm.

Liposomes containing 0.5 mol % laurdan were prepared and further diluted with TRIS buffer to 0.3 mM in small tubes (Sapphire PCR tubes, Greiner Bio-One GmbH, Frickenhausen, Germany). The desired amount of peptide stock solution was then added to achieve concentrations between 0.01 and 0.9 mM and a final volume of 280 μL. The tubes were placed in the LISO, thermostated at 25 °C and stirred with a small magnetic stirrer. Every 10 s, three emission spectra were recorded with an integration time of 100 ms and automatically averaged. Laurdan fluorescence was monitored for at least 2 h. The fluorescence spectrum of TRIS buffer was recorded and subtracted from the measurements as a background correction.

When the polarity in the surrounding of membrane-embedded laurdan increases, the intensity maximum of the laurdan fluorescence spectrum shifts from 440 to 490 nm.^37,50^ This is often interpreted as an increase in membrane hydration and fluidity. In order to quantify properties and changes of model membranes upon the addition of antimicrobial peptides, the laurdan generalized polarization *GP* was calculated at a given peptide concentration and incubation time.
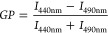
2To analyze changes in the model membrane, the difference ΔGP was determined with respect to the model membrane before peptide addition *GP*_0_.
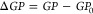
3

### 2.7 Lipid Mixing Assay

Förster resonance energy transfer (FRET) between labeled lipids can change upon membrane contact and vesicle fusion.^25,53^ In our case, double-labeled liposomes containing, both, 0.5 mol % NBD-PE (donor), and 0.5 mol % rhodamine DHPE (acceptor) labeled lipids and unlabeled liposomes were mixed together in a 1:4 (labeled/unlabeled) ratio.

The liposome suspension was further diluted with TRIS buffer in a quartz cuvette (Hellma, Müllheim, Germany) to a final concentration of 30 μM. The desired amount of peptide stock solution was added to obtain concentrations between 1 and 100 μM. With a LS 55 fluorescence spectrometer (PerkinElmer Inc., Norwalk, CT, USA), the sample was excited at 463 nm and the emission spectrum was recorded from 480 to 650 nm. The sample was stirred with a magnetic stirrer and thermostated at 25 °C. An emission spectrum was taken every 5 min for a minimum of 45 min. For reference, a final spectrum was recorded after the addition of 18 μL Triton X-100.

From the maximum intensities of NBD at 520 nm and rhodamine at 580 nm, the intensity ratio *R* was calculated.
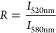
4The lipid mixing efficiency *LME* at a given peptide concentration and incubation time was then calculated according to the intensity ratio before adding the peptide *R*_0_ and after adding Triton X-100 *R*_∞_.
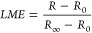
5

The maximum lipid mixing efficiency in this case is approximately 0.4. Alternatively, vesicles prepared with 1/5 of FRET labels (each 0.1% of NBD-PE and rhodamine-PE) can be used.^54^ If turbidity, light scattering, or sedimentation of liposomes and aggregates led to erratic attenuation of fluorescence spectra, the accuracy of calculations reliant on intensity maxima became compromised. Data with a decrease in NBD intensity exceeding 50% relative to the maximum intensity before peptide addition have been marked.

### 2.8 Vesicle Leakage Assay

Calcein release from liposomes was examined to characterize the permeabilization of model membranes induced by the peptides.^46,55^ For this, calcein-filled liposomes were prepared and analyzed as described in a detailed protocol.^54^

In some detail, an appropriate dry lipid film was rehydrated with an iso-osmotic calcein buffer (70 mM calcein, 10 mM TRIS, 0.5 mM EDTA, pH 7.4) and the suspension was extruded as described before. Lipid stock concentrations of 30 mM were required to compensate for losses in the subsequent procedure. The liposome suspension was loaded onto a PD-10 desalting column (GE Healthcare, Little Chalfont, United Kingdom) and eluted with TRIS buffer to exchange the external calcein buffer. In the process, fractions were collected and analyzed with regard to the ratio of entrapped and free calcein. Finally, the suitable fractions were combined and their lipid concentration determined performing a Bartlett assay.^49^ Lipid concentrations of approximately 10 mM were achieved.

The peptide stock solution was diluted with TRIS buffer in disposable polystyrene cuvettes (Sarstedt AG & Co., KG, Nümbrecht, Germany) to final concentrations between 1 and 100 μM. To start incubation, the required amount of liposome suspension was added to obtain a concentration of 30 μM. The experiment always included a reference sample with no added peptide. Cuvettes were thermostated at 25 °C and shaken with a rocking shaker (Single TEC Control Shaker, INHECO, Martinsried, Germany) at 400 rpm. Time-resolved fluorescence spectroscopy (time-correlated single photon counting TCSPC) was performed after 10 min, 30 min, 1, 2, and 5 h and fluorescence decay curves were recorded with a FluoTime 100 (PicoQuant, Berlin, Germany). A 467 nm laser diode pulsed at 1 MHz was used for excitation, while the fluorescence emission was detected at 515 nm.

For data analysis, a biexponential fit of the acquired fluorescence decay curves was performed using TimeHarp 260 software (PicoQuant, Berlin, Germany). This disclosed the amount of entrapped calcein *B*_E_ with a corresponding fluorescence lifetime τ_E_ as well as the amount of free calcein *B*_F_ with τ_F_.
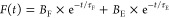
6

The total leakage *L*_total_ at a given peptide concentration and incubation time was calculated accounting for the amount of free calcein in the reference sample *B*_F0_ and effects that occur at high concentrations of entrapped calcein denoted *Q*_stat_.
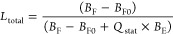
7

When the measurement was undisturbed, the denominator (called *Sum of B*) remained constant. If turbidity, light scattering, or sedimentation of liposomes and aggregates led to a decrease in fluorescence intensity, thereby compromising the accuracy of further calculations and data analysis, this resulted in a decrease in the *Sum of B*.^46^ We used the *Sum of B* as a measure for data quality^46^ and marked data with a decrease of more than 20%.

## 3 Results

### 3.1 Monolayer Experiments: Preferential Adsorption of Peptides to Charged Lipid Monolayers

The objective of this study is to examine antimicrobial peptides. We focus on antimicrobial peptides whose mechanism of action is known to involve the bacterial cell membrane.^33,40^ A lipid monolayer formed at the buffer–air interface is used to assess the adsorption and insertion of the peptides to various types of lipid monolayers.^8,56^

**Figure 1:**
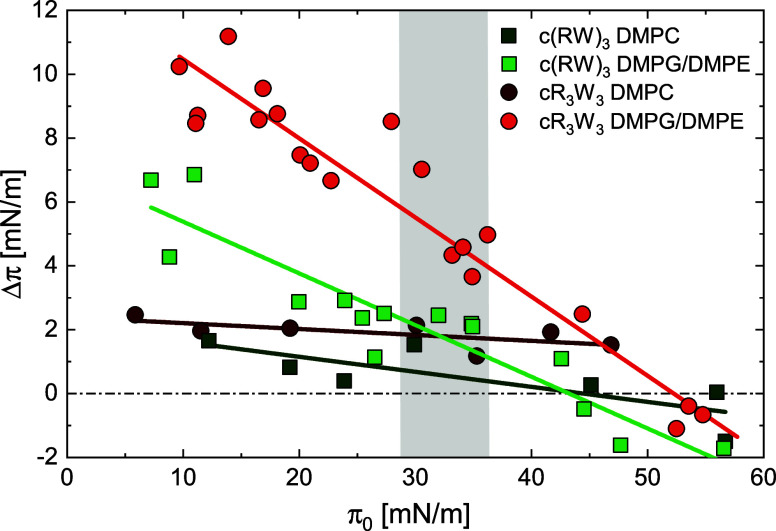
Absorption measurements characterize the adsorption and insertion of antimicrobial peptides into lipid monolayers. Changes in the surface pressure, Δπ, of a lipid monolayer 35 min after adding the peptide solution to the subphase (final concentration 900 nM) as a function of the initial surface pressure π_0_. (Dark green squares) c(RW)_3_ adsorption to DMPC monolayers, (light green squares) c(RW)_3_ adsorption to DMPG/DMPE (1:1) monolayers, (dark red dots) cR_3_W_3_ adsorption so DMPC monolayers, (light red dots) cR_3_W_3_ adsorption to DMPG/DMPE (1:1) monolayers. (10 mM TRIS; 110 mM NaCl; 0.5 mM EDTA; pH 7.4; 20 °C) The corresponding measured surface pressures π over time are provided in Figure SI1.

The peptides have an amphipathic structure that enables surface activity at the buffer–air interface, even in the absence of a lipid monolayer. Consequently, a peptide subphase concentration of 900 nM was determined to have no measurable surface activity and was used for all experiments. The peptide solution was injected to the subphase of stable lipid monolayers with an initial pressure π_0_ and the resulting change in surface pressure Δπ due to the adsorption and insertion of the peptide was determined. Figure 1 shows Δπ as a function of π_0_ for PC and PG/PE monolayers in the presence of cR_3_W_3_ and c(RW)_3_ after 35 min (corresponding time traces provided in Figure SI1). At an initial pressure π_0_ of 30 to 35 mN/m (shaded gray in Figure 1), the behavior of lipids in the monolayer is similar to that of lipids in the bilayer of model or cell membranes.^57^

An increase in surface pressure after injection of the peptide solutions was observed in most cases, giving positive Δπ values. The smaller the initial surface pressure π_0_, the higher the changes in surface pressure Δπ. This indicates that the peptides not only adsorb to the monolayer, but also insert into the lipid monolayer. This is typically driven by the hydrophobic effect.^8,58^

For zwitterionic PC monolayers, only small changes in surface pressure of less than 3 mN/m were observed (dark red dots and dark green squares in Figure 1). Negatively charged PG/PE monolayers generally resulted in higher increases in surface pressure in the presence of the peptides (light red dots and light green squares in Figure 1).

In contrast to mammalian cell membranes, bacterial cell membranes contain a considerable proportion of negatively charged lipids.^47^ The different membrane charges in bacterial or mammalian membranes are represented here by the zwitterionic PC monolayer and PG/PE monolayer that is partially negatively charged, respectively. Junkes *et al.* have demonstrated a certain selectivity, especially for c(RW)_3_, for bacterial over eukaryotic cells.^33^ As expected and consistent, both antimicrobial peptides showed a stronger adsorption to charged lipid monolayers compared to zwitterionic ones.

When comparing the two peptides for a given lipid monolayer and initial surface pressure, cR_3_W_3_ always caused the greater increase in surface pressure. For example, considering a PG/PE monolayer with an initial pressure of 35 mN/m, the change in surface pressure for c(RW)_3_ was approximately 2 mN/m and for cR_3_W_3_ about twice as much, approximately 4 mN/m. The antimicrobial activity data (MIC) of the two peptides also show this ratio: the *E. coli* MIC determined for cR_3_W_3_ was 11 and 23 μM for c(RW)_3_.^33^ The more pronounced insertion observed for cR_3_W_3_ is therefore not only consistent with the antimicrobial activity, but also with the more pronounced hydrophobicity and amphipathicity of the molecule. Based on monolayer data, it remains unclear whether this increased insertion is attributable to a higher number of adsorbed peptides, a higher surface area per peptide, or a variation in insertion geometry. Tryptophan fluorescence revealed a deeper insertion into the lipid bilayer of cR_3_W_3_ compared to c(RW)_3_.^33^

In summary, both peptides demonstrated a preference for charged PG/PE monolayers over PC monolayers, indicating a promising prerequisite for selectivity. We observed a stronger adsorption and insertion of cR_3_W_3_ in lipid monolayers compared to c(RW)_3_. This highlights the more pronounced hydrophobicity of cR_3_W_3_.^8,58^ The alternating arrangement of R and W residues in c(RW)_3_ results in a smaller continuous hydrophobic surface and less interaction with the lipid acyl chains. Thus, membrane insertion scales with the continuous hydrophobic surface of the peptide.

### 3.2 Isothermal Titration Calorimetry (ITC): Binding of Peptides to Model Membranes is Consistent with Their Antimicrobial Activity and Selectivity

**Figure 2:**
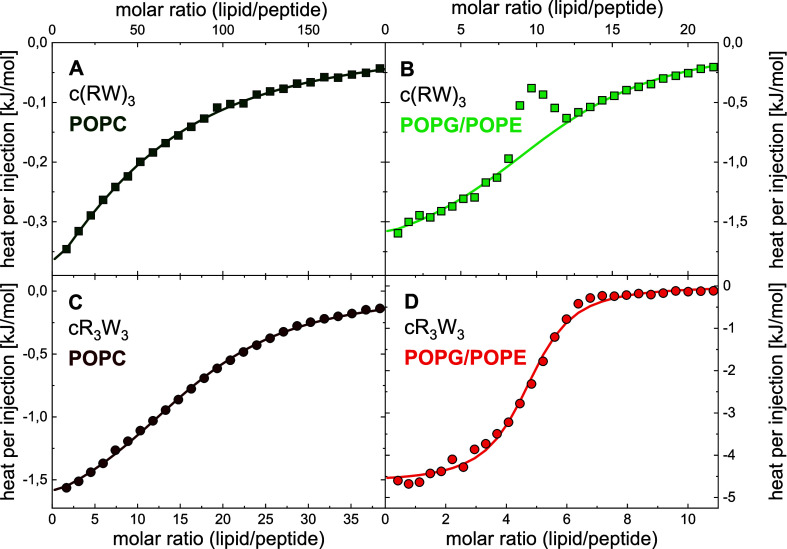
Isothermal titration calorimetry analysis of the interaction of antimicrobial peptides with model membranes. (A) 20 mM POPC liposome suspension was titrated into 0.02 mM c(RW)_3_ solution, (B) 10 mM POPG/POPE (1:1) liposome suspension was titrated into 0.1 mM c(RW)_3_ solution, (C) 20 mM POPC liposome suspension was titrated into 0.1 mM cR_3_W_3_ solution, (D) 5 mM POPG/POPE (1:1) liposome suspension was titrated into 0.1 mM cR_3_W_3_ solution. Dots and squares represent the integrated heat per injection, lines the fit curve. The corresponding fitting parameters are summarized in Table 1. (10 mM TRIS; 110 mM NaCl; 0.5 mM EDTA; pH 7.4; 25 °C) The depicted data is representative of several experiments with slightly varied initial concentrations. Data in panel D has been published before and is reproduced here from ref (46) with permission from the Royal Society of Chemistry. Raw thermograms are provided in Figure SI2.

Isothermal titration calorimetry (ITC) measurements assess the heat response arising from the antimicrobial peptides binding to model membranes.^59,60^ POPC or POPG/POPE liposomes were titrated into the peptide solutions as indicated. This characterizes the total binding of the peptides to lipid bilayers. The heat per injection was obtained by integrating the exothermal heat response as a function of the lipid/peptide ratio (Figure SI2). Figure 2 depicts the overall sigmoidal curves that are obtained. A one-set-of-sites model was fitted to the isotherms for calculating the stoichiometry (lipid/peptide) *n*, the total binding constant *K*, and the molar binding enthalpy Δ*H*. The results are summarized in Table 1.

**Table 1:** Estimation of Thermodynamic Parameters for the Lipid–Peptide Interaction Obtained From ITC Measurements Shown in Figure 2, Stoichiometry (Lipid/Peptide)*n*, Total Binding Constant*K*, Molar Binding Enthalpy Δ*H*

lipid−peptide	*n*	*K* 10^3^ [M^−1^]	Δ*H* [kJ/mol]
(A) POPC−c(RW)_3_	47	0.79	−0.9
(B) POPG/POPE−c(RW)_3_	11	4.9	−1.9
(C) POPC−cR_3_W_3_	16	2.6	−2.0
(D) POPG/POPE−cR_3_W_3_	4.7	84	−4.7

The most pronounced interaction was observed when cR_3_W_3_ bound to the PG/PE model membrane (Figure 2D). The average number of lipids per bound peptide was 4.7 with a total binding enthalpy Δ*H* of −4.7 kJ/mol. The titration of cR_3_W_3_ with PC model vesicles revealed less binding (Figure 2C). There was no pronounced sigmoidal shape, and the determined binding constant was about 30 times lower compared to PG/PE. As observed previously, cR_3_W_3_ bound more efficiently to negatively charged PG/PE than to zwitterionic PC membranes. This is consistent with the results of the monolayer studies and strongly suggests selectivity is in part caused by differences in binding to lipid membranes.

The same binding selectivity for negatively charged model membranes over zwitterionic model membranes was found for c(RW)_3_ (Figure 2A,B). Here, another peculiarity occurred in the interaction of this peptide with the POPG/POPE liposomes. Approximately at the inflection point of the curve, some of the integrated heats deviate from the expected curve. This was described before for DPPG/DPPE liposomes by Finger *et al.* and was discussed as release of curvature stress or permeabilization of the liposomes.^40^

A comparison of the two peptides reveals that cR_3_W_3_ interacts more intensely with the model membranes compared to c(RW)_3_. Considering the calculated binding enthalpy Δ*H* for a given model membrane, we found that cR_3_W_3_ always achieved at least twice the effect of c(RW)_3_, as in the monolayer adsorption measurements. For PG/PE liposomes, this meant Δ*H* = −1.9 kJ/mol for c(RW)_3_ and Δ*H* = −4.7 kJ/mol for cR_3_W_3_. The overall binding constant of cR_3_W_3_ is increased by a factor of 4 (POPC) and 18 (POPG/POPE) compared to c(RW)_3_. This is well reflected by the higher hydrophobicity and antimicrobial activity of cR_3_W_3_ compared to c(RW)_3_.^33^

### 3.3 Laurdan Fluorescence Spectroscopy: Peptides Modulate Lipid Order in Charged Model Membranes

**Figure 3:**
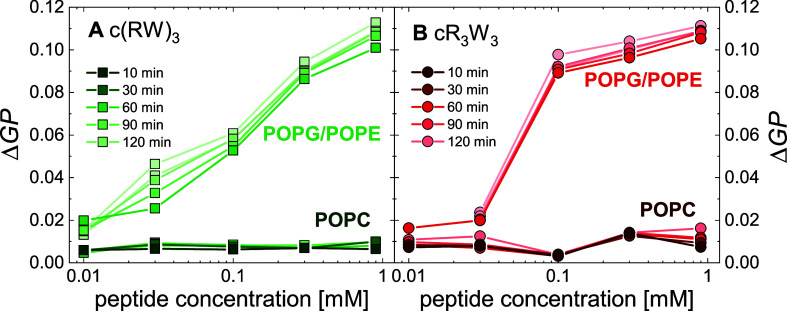
Laurdan fluorescence spectroscopy provides insight into lipid headgroup packing and membrane fluidity. This is expressed as the general polarization *GP*, which is calculated from the emission spectra provided in Figure SI3. Changes in membrane order, Δ*GP*, of 0.3 mM liposome suspensions over 2 h are shown as function of peptide concentration. (A) c(RW)_3_ was added to POPC and POPG/POPE (1:1) liposomes, (B) cR_3_W_3_ was added to POPC and POPG/POPE (1:1) liposomes. (10 mM TRIS; 110 mM NaCl; 0.5 mM EDTA; pH 7.4; 25 °C).

The fluorescent dye laurdan is sensitive to the polarity of its direct environment. Changes cause characteristic shifts in the emission spectrum of the dye. Therefore, laurdan is often used to assess lipid packing, hydration or phase transitions of lipid bilayers.^50,52,61^ The carbonyl group and hydrocarbon chain of laurdan place the dye close to the hydrophobic part of a membrane. Its fluorophore remains at the level of the glycerol backbone close to the lipid headgroups.^62^ In particular, the packing of lipid headgroups and their hydration are probed in terms of the general polarization *GP*. Δ*GP* quantifies the changes induced by the peptides. Laurdan was incorporated into liposomes, and POPC and POPG/POPE liposomes were then incubated with the antimicrobial peptides for up to 2 h. We monitored the general polarization in relation to the peptide concentration and incubation time (Figure 3).

In POPC liposomes, only minor changes were observed for cR_3_W_3_ and c(RW)_3_ (Figure 3A dark green squares and Figure 3B dark red dots). This is in line with our expectations, as we found only weak adsorption and binding of the peptides to PC model membranes.

When cR_3_W_3_ and c(RW)_3_ were added to POPG/POPE liposomes, immediate changes occurred within the first minutes (Figure 3A light green squares and Figure 3B light red dots). ΔGP increased with peptide concentration and only slightly with incubation time. This indicates a decreasing polarity of laurdan’s environment and lipid headgroup hydration. Binding of antimicrobial R- and W-rich peptides to the negatively charged PG/PE model membrane appears to increase the packing density of lipid headgroups in the lipid bilayer, similar to the increase in surface pressure observed in the lipid monolayer. NMR studies on cWFW indicated that the peptides orient at the interface between the lipid headgroups and the fatty acid chains owing to their amphipathic nature.^45^

In conclusion, in accordance with their binding behavior and antimicrobial selectivity, we observed a more pronounced change in lipid headgroup packing and hydration in charged PG/PE model membranes due to peptide binding compared to PC model membranes.

Finger *et al.* thoroughly characterized the ability of the whole family of cyclic peptides to cluster PG lipids out of PG/PE mixed membranes.^40,41^ One could speculate that lipid clustering might be another manifestation of the changes in lipid headgroup packing that also change the environment of laurdan.

### 3.4 Dynamic Light Scattering (DLS): Peptides Induce Aggregation of Charged Vesicles

**Figure 4:**
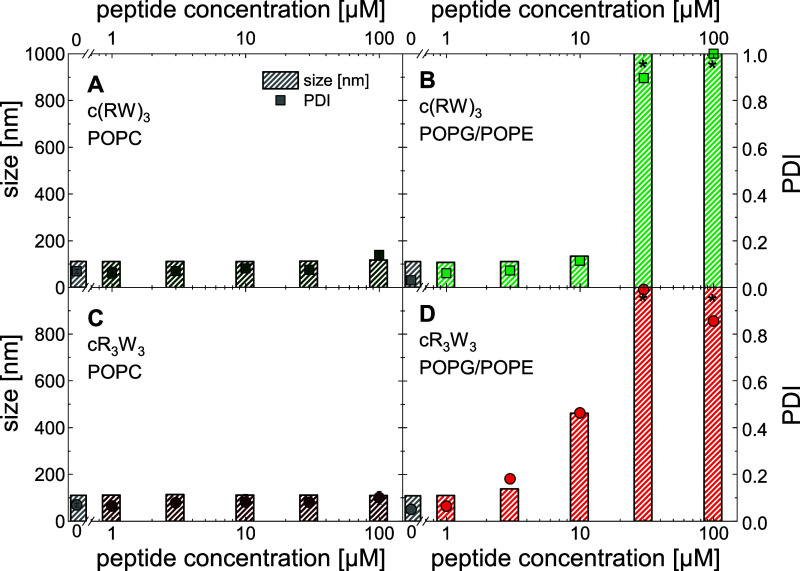
Dynamic light scattering (DLS) analysis reveal the particle size (bars) and size distribution PDI (dots) of 30 μM liposome suspension after incubation with various concentrations of antimicrobial peptides for 24 h. Asterisks mark a particle sizes >1000 nm. (A) POPC liposomes and (B) POPG/POPE (1:1) liposomes were incubated with c(RW)_3_. (C) POPC liposomes and (D) POPG/POPE (1:1) liposomes were incubated with cR_3_W_3_. (10 mM TRIS; 110 mM NaCl; 0.5 mM EDTA; pH 7.4; 25 °C) Data in panel D has been published before and is reproduced here from ref (46) with permission from the Royal Society of Chemistry.

We have previously described that incubating POPG/POPE liposomes with cR_3_W_3_ leads to visible aggregation above a certain peptide concentration.^46^Figure 4 summarizes the particle size and size distribution obtained by DLS measurements for 30 μM either POPC or POPG/POPE liposomes incubated with antimicrobial peptides for 24 h.

We consistently observed no appreciable change in particle size with any peptide concentration added to zwitterionic PC vesicles (Figure 4A,C). The corresponding size distribution, expressed as PDI, was also constant over the concentration range considered.

When cR_3_W_3_ was added to PG/PE vesicles (Figure 4D), first changes were detected at 3 μM peptide concentration. The particle size and the size distribution increased as the concentration of cR_3_W_3_ was further increased. In the presence of c(RW)_3_, PG/PE vesicles showed similar behavior (Figure 4B). However, the increase in particle size and size distribution required higher c(RW)_3_ peptide concentrations between 10 and 30 μM.

The induced vesicle aggregation can be attributed to the electrostatic stabilization of the colloidal liposome suspension by the negative surface charge of POPG/POPE liposomes that is abolished upon neutralization by the bound peptides.^63^ This enables membrane contact between liposomes and the formation of larger aggregates. Theoretically, the charge neutrality is achieved at a lipid/peptide molar ratio of 6:1 (12:1 without leakage, only bound to the outer leaflet), which corresponds to an added peptide concentration of 5 μM (2.5 μM without leakage, only bound to the outer leaflet) in this experimental setup. We could even speculate that the bound peptides cross-connect adjacent vesicles. In contrast, POPC liposome suspensions, being zwitterionic with a neutral net charge, exhibit no tendency to aggregate upon additions of peptides (Figure 4A,C).

In summary, PC model membranes showed no signs of membrane contact, vesicle fusion or aggregation in the presence of the antimicrobial peptides studied. However, PG/PE model membranes showed these phenomena. For both peptides, membrane contact and vesicle aggregation were observed. Therefore, we assume that vesicle fusion can also occur.^46^

### 3.5 Lipid Mixing Assay: Peptides Promote Membrane Contact of Charged Vesicles

**Figure 5:**
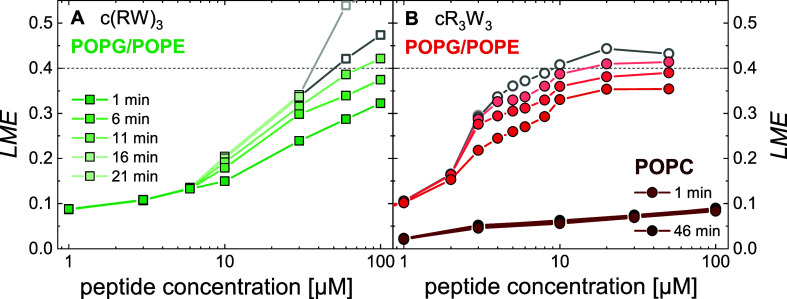
Lipid mixing efficiency *LME* of 30 μM liposome suspension after incubation with various concentrations of antimicrobial peptides. (A) POPG/POPE (1:1) liposomes (light green) were incubated with c(RW)_3_. (B) POPC liposomes (dark red) and POPG/POPE (1:1) liposomes (light red) were incubated with cR_3_W_3_. Data with poor intensity and therefore of questionable reliability are depicted in gray. (10 mM TRIS; 110 mM NaCl; 0.5 mM EDTA; pH 7.4; 25 °C) POPG/POPE data in panel B has been published before and is reproduced here from ref (46) with permission from the Royal Society of Chemistry. The corresponding emission spectra are provided in Figure SI4.

We performed FRET-based experiments using NBD-labeled and Rhodamine-labeled lipids. Double-labeled and unlabeled liposomes were mixed together and incubated with the antimicrobial peptides. When different vesicles come into close contact and fuse, the FRET behavior and the emission spectrum change (Figure SI4). The vesicle aggregation described above is already an indication that membrane contact between liposomes occurs in certain cases. The FRET assay can in particular detect the exchange of lipids upon close contact, hemifusion or fusion of liposomes.^25,53^ This is summarized and calculated as lipid mixing efficiency *LME*, and the results are shown in Figure 5.

For PC vesicles, there were no clear signs of lipid mixing, membrane contact, or vesicle fusion at any incubation time or cR_3_W_3_ peptide concentration. Overall, only *LME* values of approximately 0.1 were determined. This met our expectations, as we have observed no changes of the particle size and size distribution of PC vesicles upon the addition of peptides by DLS.

Conversely, we expected potential lipid mixing in PG/PE vesicles based on the DLS measurements. The incubation with c(RW)_3_ apparently led to a steady increase of *LME* with increasing peptide concentration and incubation time (Figure 5A). At higher peptide concentrations, the theoretical maximum for the current experimental parameters of approximately 0.4 was reached.^25^ This indicates irregular fluorescence intensities caused by large particles. Within the first minutes, changes were detected at a peptide concentration of 10 μM or higher. At incubation times of more than 20 min and peptide concentrations that had been found to cause vesicle aggregation, light scattering and other artifacts caused a severe decrease of the measured intensity. This data is not reliable and is therefore depicted gray in Figure 5. As vesicle fusion is supposedly occurring and finishing within the first 10 min of incubation, longer times are increasingly influenced by changes, such as vesicle aggregation and were not considered here.

The incubation of PG/PE vesicles with cR_3_W_3_ induces immediate changes in *LME* within minutes (Figure 5A light red dots). An increase in *LME* was observed at peptide concentrations starting as low as 3 μM. This was followed by an increase in *LME* with increasing peptide concentration, reaching a maximum plateau of 0.4 at approximately 10 μM.^46^ Again, as the incubation time increased, so did the *LME* and the uncertainty of the results.

In addition to the DLS results described in the previous paragraph, we can now state that the peptides promote membrane contact in PG/PE model membranes with lipid exchange, hemifusion, or full fusion of vesicles. This occurred rapidly after binding of the peptides and correlates with their antimicrobial activity. Again, PC model membranes showed no signs of membrane contact or vesicle fusion in the presence of the antimicrobial peptides.

### 3.6 Vesicle Leakage: Peptides Induce Membrane Permeabilization by Different Mechanisms

**Figure 6:**
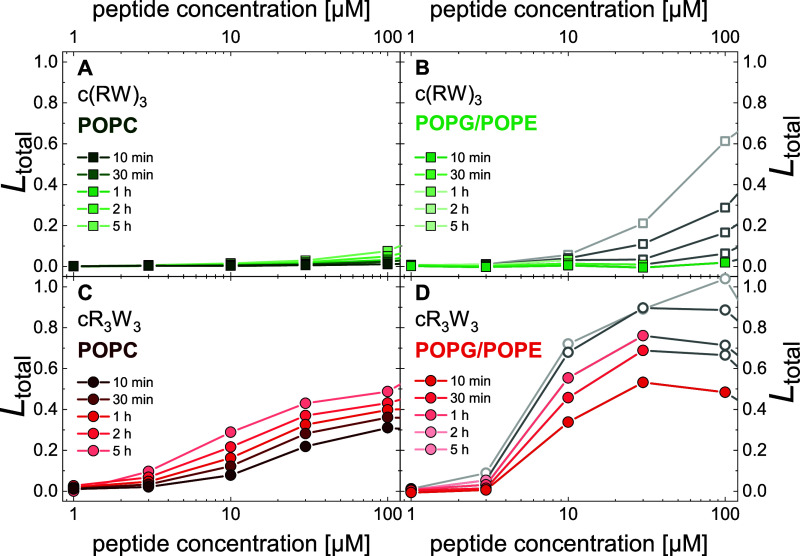
Calcein leakage reveals the permeabilization behavior of antimicrobial peptides. The total vesicle leakage *L*_total_ of 30 μM liposome suspensions is shown as a function of peptide concentration at increasing incubation times. (A) POPC liposomes were incubated with c(RW)_3_, (B) POPG/POPE (1:1) liposomes were incubated with c(RW)_3_, (C) POPC liposomes were incubated with cR_3_W_3_, (D) POPG/POPE (1:1) liposomes were incubated with cR_3_W_3_. As illustrated in Figure SI6, data with a decrease in *Sum of B* of more than 20% is depicted in gray. (10 mM TRIS; 110 mM NaCl; 0.5 mM EDTA; pH 7.4; 25 °C) Data in panel D has been published before and is reproduced here from ref (46) with permission from the Royal Society of Chemistry.

The vesicle leakage assay, as conducted here, determines the calcein leakage from liposomes in the presence of the antimicrobial peptides.^54,55^ Based on the estimated total leakage *L*_total_, we assess the membrane permeabilization behavior of the peptides. Figure 6 shows the results for POPC and POPG/POPE liposomes after incubation with various concentrations of cR_3_W_3_ and c(RW)_3_ over an incubation time of up to 5 h. Parameters of the biexponential fits to the decay curves are given in Tables SI1—SI4.

#### 3.6.1 cR_3_W_3_ Induces Leaky Fusion and Fast Vesicle Leakage

Previously, we have discussed the leakage behavior of cR_3_W_3_ on calcein-filled POPG/POPE liposomes and the challenges of interpreting such data in terms of reliable quantification and the likelihood of concomitant or related vesicle fusion.^46^Figure 6D contains the characteristic sigmoidal curve obtained when *L*_total_ is shown as a function of peptide concentration. Starting from a peptide concentration of 3 μM, we obtained fast vesicle leakage within the first 10 min of the incubation. Vesicle leakage increased rapidly with peptide concentration, reaching *L*_total_ ≈ 50% at 30 μM cR_3_W_3_ after 10 min. This is caused by an all-or-none leakage behavior.^46^ We also found and discussed earlier that leaky fusion is an essential part of the leakage mechanism.^46^ As the incubation time increases, *L*_total_ increased along with the uncertainty of the quantification caused by large particles. This led to a substantial decrease in *Sum of B* and the affected data is depicted in gray in Figure 6. We must assume that in these cases, the apparent leakage is potentially overestimated. Nevertheless, we can conclude that cR_3_W_3_ most likely induces leaky fusion that manifests as fast vesicle leakage in PG/PE model membranes.

The incubation of POPC liposomes with increasing concentrations of cR_3_W_3_ resulted in less leakage activity compard to POPG/POPE (1:1) liposomes in general (Figure 6C). We again found fast leakage from 3 μM, which increased steadily with peptide concentration. After 10 min, 30 μM cR_3_W_3_ achieved *L*_total_ ≈ 20%. The leakage activity increased with incubation time, but in contrast to PG/PE liposomes, no changes in *Sum of B* were observed. Also in POPC, cR_3_W_3_ induces all-or-none leakage (Figure SI5).

In summary, PC vesicles showed no signs of membrane contact, vesicle fusion or aggregation in the presence of cR_3_W_3_. Therefore, it can be assumed that the acquired leakage data are reliable and unbiased by artifacts. Furthermore, leaky fusion can likely be excluded as a potential leakage mechanism. Thus, we can conclude that cR_3_W_3_ induces limited and fast vesicle leakage in PC model membranes by a mechanism different from vesicle fusion. If fast and annealing leakage, as caused by cR_3_W_3_ in POPC vesicles, is not related to fusion, it is supposed to be caused by asymmetry stress.^64−66^ This leakage mechanism is caused by the hydrophobically driven insertion of the peptide initially only into the outer leaflet of a membrane. Apparently, the limited insertion of cR_3_W_3_ into PC model membranes, quantified in the monolayer experiments, is sufficient.

#### 3.6.2 c(RW)_3_ Induces less, Slow Vesicle Leakage

Although we have previously shown a weak binding of c(RW)_3_ to POPC liposomes, we could not detect any appreciable leakage activity (Figure 6A).

In POPG/POPE liposomes, c(RW)_3_ potentially caused some leakage but only slow vesicle leakage (Figure 6B) and less leakage activity compared to cR_3_W_3_. No fast vesicle leakage was observed after 10 min. Again, data quality of longer incubation times is affected by large particles, which is reflected in a significant decrease in *Sum of B*. The affected data is marked in gray in Figure 6.

Therefore, the leakage mechanism is unlikely to be either asymmetric stress or fusion related, even though, c(RW)_3_ did show signs of membrane contact and vesicle fusion in the relevant time span of up to 10 min. Interpretation of the calcein leakage from POPG/POPE liposomes in the presence of c(RW)_3_ was also limited by a decrease in *Sum of B* corresponding to the increase in vesicle aggregation. The apparent leakage obtained for higher peptide concentrations and longer incubation times could be overestimated or even nonexisting. However, we cannot exclude the possibility that c(RW)_3_ induces some slow vesicle leakage in PG/PE model membranes.

#### 3.6.3 Correlation between Different Leakage Mechanisms and Selectivity

Comparing both peptides, we can conclude that the leakage activity of the peptides corresponds to their hydrophobicity, binding behavior, and antimicrobial activity in cells.^33^ The more active peptide, cR_3_W_3_, causes more pronounced membrane permeabilization in model membranes at lower concentrations. Furthermore, we can show that the two peptides differ in their leakage mechanism. Only cR_3_W_3_ induces leaky fusion and asymmetry stress in POPC, whereas c(RW)_3_ generally shows barely any leakage activity, especially not for PC model membranes.

Furthermore, the leakage characteristics of the peptides described here may also be an explanation for their differences and peculiarities in ITC measurements. A deviation from the expected sigmoidal curve occurs only for c(RW)_3_. Finger *et al.* hypothesize that at a certain lipid/peptide ratio, there is a significant permeabilization of the liposome membrane, which then results in a sudden binding of the peptide to the inner leaflet of the liposomes, thereby causing the observed deviation.^40^ In our case, leakage would stop when the added vesicles increases above a threshold. The described leakage activity only at higher c(RW)_3_ peptide concentrations support this hypothesis. At the same time, the pronounced, fast vesicle leakage mechanism for cR_3_W_3_ would explain the absence of deviations from the expected ITC curve (Figure 2). Even at low lipid/peptide ratios, liposome membrane permeabilization by cR_3_W_3_ occurs quickly and peptides bind equally to the outer and inner liposome leaflets. Consequently, the system is always balanced, exhibiting no abrupt changes in the ITC titration data.

The onset of increase in particle size, changes in lipid packing (*GP*), lipid mixing, and leakage are coinciding with charge neutralization (*R*_c_ = 1, Figure SI7). Increases in particle size and lipid mixing already start when the peptide concentration suffices to neutralize the outer membrane leaflet.

## 4 Discussion

Membrane permeabilization behavior is often used to investigate and explain the antimicrobial activity of membrane-active peptides.^8,67,68^ However, the antimicrobial peptides used here target in particular the bacterial membrane, but permeabilization is not the predominant mechanism of action.^33,38^ Our aim was rather to elucidate the differences in selectivity between the two small cyclic peptides, which differ only in the arrangement of their charged and aromatic side chains. For this, we examined their effects on zwitterionic PC and negatively charged PG/PE model membranes, which are the most abundant phospholipids in either mammalian or bacterial cell membranes.^47,48^ In general, we could confirm previous findings and expectations for the two cyclic antimicrobial peptides, even though the membrane model system we use is a highly simplified representation of the lipid part of cell membranes. In particular the concentrations for induction of leakage and other perturbations are in agreement with the reported differences in antimicrobial activity and selectivity of the individual peptides. cR_3_W_3_ is antimicrobial at lower concentrations (MIC 11 μM), but also more cell toxic and hemolytic than c(RW)_3_ (MIC 23 μM).^33^ Thus, cR_3_W_3_ proved to be less selective than either c(RW)_3_ or cWFW.

To investigate the effects of the antimicrobial peptides on model vesicles, we primarily focused on an incubation time of 10 min. This duration provides sufficient time for the relevant interactions to occur while minimizing artifacts that may arise from longer incubation periods, such as larger vesicle aggregates that interfere with optical measurements. Therefore, spectroscopic data obtained from potentially aggregated or fused vesicles are marked and must be interpreted with caution. Conclusions drawn from these measurements should be considered carefully. Longer incubation times can reveal processes that (re)occur stochastically or require nucleation, rare orientations, or conformations. Monolayer adsorption, however, typically requires longer time for equilibration.

Let us also consider the consistency of the various methods used to examine vesicles. Figure SI7 compares lipid and peptide concentrations across the different experiments on vesicles presented in this report for cR_3_W_3_ interacting with POPG/POPE (1:1) vesicles. The molar ratio (MR) as well as the charge neutralization conditions for the entire sample (*R*_c_ = 1), or only the lipids in the outer membrane leaflet (*R*_c,outer_ = 1), are marked by dotted lines. As previously reported and discussed for an antimicrobial polymer,^54^ no pronounced leakage, lipid mixing, or aggregation are observed below the potential charge neutralization of the outer membrane leaflet by the added peptide (vertical line in Figure SI7). An increase in particle size and apparent lipid mixing begins at *R*_c,outer_ > 1, and leakage, as well as changes in laurdan fluorescence, appear to require higher peptide-to-lipid ratios in the sample. The apparent stoichiometry of binding, as determined by ITC, coincides with *R*_c_ = 1, indicating charge neutralization involving both the outer and inner membrane leaflets, as expected for leaky vesicles. Therefore, electrostatic interactions seem to drive the effect of cR_3_W_3_ on membranes. For c(RW)_3_ (under identical experimental conditions as for cR_3_W_3_, though the onset of observed effects differs, not shown), the binding stoichiometry to POPG/POPE (1:1) also coincides with the neutralization of the outer membrane leaflet, in agreement with the negligible leakage activity and reduced lipid mixing activity. The differing apparent binding affinities of the peptides to POPG/POPE (1:1) lead to the sigmoidal curve observed for cR_3_W_3_ and the more continuous curve for c(RW)_3_ in Figure 2D,B, respectively. The varying extent of binding likely results in the sigmoidal or more continuous increase in secondary effects (lipid mixing, laurdan fluorescence, increase in particle size, leakage) as a function of peptide concentration observed in the other methods.

Selectivity in membrane-activity can be caused by differences in binding to lipid membranes or differences in extent and type of membrane damage.^7−9,68^ Here, we will discuss (I) different membrane binding as function of lipid composition and secondary effects. En route, the mechanism of antibacterial activity previously investigated in bacteria is corroborated by our more detailed model studies. (II) We will argue that differences between the peptides and in particular their membrane permeabilization mechanisms can explain selectivity. (III) We finally put forward a balance of leakage and other effects that results in the differing selectivities.

### 4.1 Membrane Binding Requires Charged Lipids and Leads to Diverse Secondary Effects including Changes in Lipid Packing and Membrane Leakage

The peptides cR_3_W_3_ and c(RW)_3_, both, bind to model membranes with a preference for negatively charged PG/PE membranes over zwitterionic PC membranes. In particular, binding of cR_3_W_3_ to PG/PE model membranes is very similar to PG/PC,^46^ indicating that the PE headgroup does not contribute specifically to the binding. Only for the charged model membranes, a substantial adsorption and insertion is found in monolayer experiments and with liposomes (Figures 1 and 2). This, in turn, leads to secondary effects predominantly in negatively charged membranes, namely leakage, changes in lipid headgroup packing, and enhanced membrane contact.

In more detail, first, we found the peptides to induce leakage in model membranes (Figure 6, discussed below). Wenzel *et al.* and Scheinpflug *et al.* examined the influence of the related linear peptide RWRWRW and selective cyclic peptide cWFW on bacterial membranes in more detail^36−38^ and excluded direct membrane permeabilization as the mechanism of antimicrobial action. We will discuss below the potential role of leakage in model membranes for selectivity.

Second, investigating further consequences of peptide binding using laurdan, we found an increase in the order of the lipid headgroups in the PG/PE model membranes, but not in PC model membranes (Figure 3). Interestingly, Scheinpflug *et al.* found a similar effect on laurdan induced by cWFW in model and bacterial membranes^37^ and discuss this altered membrane packing as central to the mechanisms of action they observed in bacteria, namely the dissociation of essential membrane proteins, the inhibition of cell wall synthesis, and autolysis. Based on our findings in model membranes, we assume that cR_3_W_3_ and c(RW)_3_ also act via the effects confirmed in bacteria, namely alterations in lateral packing that culminate in the dissociation of membrane proteins, among other effects. Importantly, this cascade of effects appears to be selective to the lipid composition, as our comparison of PC and PG/PE model membranes suggests.

Third, the peptides induce membrane contact, *i.e.* vesicle aggregation (Figure 4) and fusion (Figure 5) only for the negatively charged PG/PE model membranes. All these pronounced differences resulting from different binding to zwitterionic and charged model membranes are typically found for antimicrobial peptides (*e.g.*^69^) and suggest also a strong selectivity for bacteria over mammalian cells.

### 4.2 Peptides Induce Different Types of Membrane Permeabilization that Account for Low or High Selectivity

In direct comparison, the two peptides differ in their binding to, both, zwitterionic and negatively charged membranes. cR_3_W_3_ generally binds and inserts more than c(RW)_3_ (Figure 1, Table 1, and tryptophan fluorescence^33^), and induces more pronounced secondary effects. If the difference were solely attributable to binding affinity, one would expect that the peptide with the lower affinity would produce the same membrane effects at double the concentration. However, this is not observed, suggesting that the peptides interact with the membrane through distinct mechanisms. Consequently, the stronger initial binding of cR_3_W_3_ to the membrane alone cannot explain its reduced selectivity. Here, we will especially mention the different types of membrane permeabilization.

When interpreting experimental leakage data that was obtained from model membranes, it is essential to distinguish different types of leakage: leakage that is probably enhanced and only possible in POPG/POPE model vesicles but not or much less relevant in microbes, such as leaky membrane fusion,^46,54^ and leakage resulting from other molecular behavior that is potentially also relevant in cells and microbes. Furthermore, light scattering or sedimentation of large particles, for example related to vesicle aggregation, can lead to largely overestimated or slightly underestimated leakage. The data we marked in Figure 6 suffers from reduced intensity (evaluated as described in^46^) and provides an upper limit of the total leakage.

As mentioned before, for the cyclic R- and W-rich hexapeptides, membrane permeabilization is unlikely to be the main mechanism of action against bacteria, but it is nevertheless important. In order to explain the different selectivities, let us first focus on zwitterionic PC model membranes. PC membranes are neither prone to leaky fusion,^70^ nor to any of the artifacts described above for charged vesicles.^46,54^ The fast leakage induced only by cR_3_W_3_ in POPC vesicles is attributed to asymmetry stress caused by the peptide initially inserting into the outer membrane leaflet only.^64−66^ This is in line with the monolayer results (Figure 1) that showed more insertion of cR_3_W_3_ compared to c(RW)_3_. Such membrane permeabilization by asymmetry stress is, thus, unselective as it relies on the insertion into membranes, driven by the hydrophobic effect irrespective of lipid headgroup composition. Asymmetry stress is not only relevant in model membranes. It could also occur in zwitterionic mammalian cell membranes and is potentially involved in the toxicity observed for HeLa cells or erythrocytes.^33^ Therefore, the fast vesicle leakage observed only for cR_3_W_3_ may account for the reduced selectivity of cR_3_W_3_ and other peptides with blocks of three or more W residues or other hydrophobic subunits or side chains compared to c(RW)_3_, cWFW, and other peptides with W residues scattered in the sequence.^11^

The role of leakage caused in negatively charged POPG/POPE vesicles for antimicrobial activity is less clear. As we have discussed before, cR_3_W_3_ probably causes permeabilization of PG/PE vesicles by leaky fusion.^46^ However, if this type of leakage is directly relevant to bacteria is questionable. Other types of leakage, such as asymmetry stress or pore formation, might occur additionally.

The more selective c(RW)_3_ requires higher peptide concentrations for leakage in PG/PE. In particular, in the concentration range most relevant for antimicrobial activity,^71^ c(RW)_3_ causes no fast leakage, *i.e.* neither asymmetry stress nor leaky fusion.^65^ We assume that c(RW)_3_ causes no or only little membrane permeabilization in bacteria or mammalian cells at μM peptide concentration. From our model studies, we cannot conclude about its ability to penetrate cell membranes without leakage, *i.e.* its potential as CPP.

### 4.3 Favorable Balance of Different Types of Membrane Damage is Advantageous While Efficient Membrane Leakage Reduces Selectivity

Now, let us briefly summarize the probable contribution of membrane permeabilization activity to biological selectivity and how the balance with other membrane perturbation effects might explain differences in peptide selectivity. For antimicrobial activity of R- and W-rich hexapeptides, changes in membrane packing and their impairment of proper membrane protein location seem sufficient.^36−38^ In the slightly less active peptides (for example c(RW)_3_ and probably also linear RWRWRW and cWFW), these subtle changes in membrane packing seems to account for activity and the absence or limitation of leakage ensures good selectivity.

Fast and efficient membrane permeabilization presumably by asymmetry stress, on the other hand, is a likely but nonselective mechanism of action in zwitterionic and charged membranes only for the more hydrophobic cR_3_W_3_ and other highly hydrophobic compounds in general.^8,21−25^ In drug delivery, there is a similar toxicity-efficiency dilemma.^26−28^ The unselective leakage relies on a pronounced insertion of the cR_3_W_3_ into membranes that can be attributed to the large hydrophobic molecular surface with three adjacent W residues (Figures 1 and 2, confirmed by retentions times and circular dichroism spectroscopy^33^). Additionally, a deep insertion might counteract efficient membrane translocation in the context of CPPs.^20^

In conjunction with similar previous findings in R- and W-rich peptides,^11^ we propose to avoid such sequences in the design or selection of membrane active antimicrobials and other membrane-active compounds, such as CPPs.

## 5 Conclusion

The two R- and W-rich peptides investigated here were proposed to act against bacteria via changes in membrane packing and hydration that impair peripheral membranes proteins.^36−38^ In our biophysical model studies, the peptides differ in their binding to both zwitterionic and negatively charged membranes and in their ability to induce membrane permeabilization, especially by asymmetry stress. In more detail, from the more pronounced interaction of the peptides with negatively charged PG/PE model membranes compared to zwitterionic PC model membranes, we can conclude that the negative charge of the bacterial membrane is crucial for mediating binding, a trivial prerequisite for further membrane perturbations.

Furthermore, the peptide with the larger continuous hydrophobic molecular surface (three adjacent W residues) binds and particularly inserts more into membranes and, thus, more severely affects membranes. cR_3_W_3_ does bind and insert much less in zwitterionic PC membranes compared to charged membranes, but apparently sufficiently to cause membrane permeabilization by asymmetry stress that plausibly explains the hemolytic activity and potentially the toxicity to HeLa cells. Membrane permeabilization by asymmetry stress (in zwitterionic membranes) is, thus, a likely but nonselective mechanism of action for relatively hydrophobic compounds, such as cR_3_W_3_.

For the beneficial selectivity of c(RW)_3_ and other compounds, the limited binding to PC membranes and also comparably much reduced membrane permeabilization in the μM peptide concentration range seems required. In particular, the selective peptide causes much less or no unselective leakage attributed to asymmetry stress. Induced membrane permeabilization is, thus, only potentially contributing to antimicrobial activity, while it can critically reduce selectivity.

Presumably more important for their antimicrobial activity, both peptides cause changes in lipid headgroup packing as examined by changes in laurdan fluorescence. While these changes in membrane packing seem not to correlate strongly with selectivity, they have been discussed as being important for peptide activity before.^36−38^ In particular, they are thought to be related to the dissociation of membrane proteins, alteration in cell–wall synthesis, and autolysis.^37^

In conclusion, among non-mebrane related effects, a favorable balance between the actually selective, bacteriostatic or bactericidal membrane perturbations, here observed as changes in lipid headgroup packing, and the type of membrane leakage caused by unselective membrane insertion (presumably causing asymmetry stress) seems to be required for the selectivity of antimicrobial peptides.

For the design or selection of antimicrobials and other membrane-active compounds, criteria that do not focus on maximal membrane permeabilization efficiency in model membranes are highly favorable. In antimicrobial compounds in particular, membrane permeabilization by asymmetry stress should be avoided. Improving the criteria for new membrane-active compounds is especially timely in view of high throughput approaches,^44,72^ directed evolution,^73,74^ and computer aided learning.^75,76^

## Supporting Information Available

Raw data of monolayer experiments, isothermal titration calorimetry (including analysis model), fluorescence spectroscopy of laurdan and lipid mixing efficiency, additional represenations of leakage data including details about data quality and data analysis, as well as a comparison of experimental conditions (PDF)

## References

[ref1] Murray C. J.; Ikuta K. S.; Sharara F.; Swetschinski L.; Robles Aguilar G.; Gray A.; Han C.; Bisignano C.; Rao P.; Wool E.; et al. Global burden of bacterial antimicrobial resistance in 2019: a systematic analysis. Lancet 2022, 399, 629–655. 10.1016/s0140-6736(21)02724-0. 35065702 PMC8841637

[ref2] Hancock R. E.; Sahl H.-G. Antimicrobial and host-defense peptides as new anti-infective therapeutic strategies. Nat. Biotechnol. 2006, 24, 1551–1557. 10.1038/nbt1267. 17160061

[ref3] Wimley W. C.; Hristova K. Antimicrobial peptides: successes, challenges and unanswered questions. J. Membr. Biol. 2011, 239, 27–34. 10.1007/s00232-011-9343-0. 21225255 PMC3166253

[ref4] Baltzer S. A.; Brown M. H. Antimicrobial peptides−promising alternatives to conventional antibiotics. Microb. Physiol. 2011, 20, 228–235. 10.1159/000331009. 21894027

[ref5] Browne K.; Chakraborty S.; Chen R.; Willcox M. D.; Black D. S.; Walsh W. R.; Kumar N. A new era of antibiotics: the clinical potential of antimicrobial peptides. Int. J. Mol. Sci. 2020, 21, 7047 10.3390/ijms21197047. 32987946 PMC7582481

[ref6] Magana M.; Pushpanathan M.; Santos A. L.; Leanse L.; Fernandez M.; Ioannidis A.; Giulianotti M. A.; Apidianakis Y.; Bradfute S.; Ferguson A. L.; et al. The value of antimicrobial peptides in the age of resistance. Lancet Infect. Dis. 2020, 20, e216–e230. 10.1016/S1473-3099(20)30327-3. 32653070

[ref7] Matsuzaki K. Control of cell selectivity of antimicrobial peptides. Biochim. Biophys. Acta, Biomembr. 2009, 1788, 1687–1692. 10.1016/j.bbamem.2008.09.013. 18952049

[ref8] Stulz A.; Vogt A.; Saar J. S.; Akil L.; Lienkamp K.; Hoernke M. Quantified membrane permeabilization indicates the lipid selectivity of membrane-active antimicrobials. Langmuir 2019, 35, 16366–16376. 10.1021/acs.langmuir.9b01849. 31710807

[ref9] Bobone S.; Stella L. Selectivity of antimicrobial peptides: a complex interplay of multiple equilibria. Antimicrobial Peptides: Basics for Clinical Application; Springer, 2019; Vol. 1117, pp 175–214. 10.1007/978-981-13-3588-4_11. 30980359

[ref10] Xu E.; Saltzman W. M.; Piotrowski-Daspit A. S. Escaping the endosome: assessing cellular trafficking mechanisms of non-viral vehicles. J. Controlled Release 2021, 335, 465–480. 10.1016/j.jconrel.2021.05.038. 34077782

[ref11] Khemaissa S.; Walrant A.; Sagan S. Tryptophan, more than just an interfacial amino acid in the membrane activity of cationic cell-penetrating and antimicrobial peptides. Q. Rev. Biophys. 2022, 55, e10 10.1017/s0033583522000105. 35979810

[ref12] Röckendorf N.; Nehls C.; Gutsmann T. Design of membrane active peptides considering multi-objective optimization for biomedical application. Membranes 2022, 12, 180 10.3390/membranes12020180. 35207101 PMC8880019

[ref13] Taylor S. D.; Palmer M. The action mechanism of daptomycin. Bioorg. Med. Chem. 2016, 24, 6253–6268. 10.1016/j.bmc.2016.05.052. 27288182

[ref14] Schilling N. A.; Berscheid A.; Schumacher J.; Saur J. S.; Konnerth M. C.; Wirtz S. N.; Beltrán-Beleña J. M.; Zipperer A.; Krismer B.; Peschel A.; et al. Synthetic lugdunin analogues reveal essential structural motifs for antimicrobial action and proton translocation capability. Angew. Chem., Int. Ed. 2019, 58, 9234–9238. 10.1002/anie.201901589. PMC6618241 31059155

[ref15] Ruppelt D.; Trollmann M. F.; Dema T.; Wirtz S. N.; Flegel H.; Mönnikes S.; Grond S.; Böckmann R. A.; Steinem C. The antimicrobial fibupeptide lugdunin forms water-filled channel structures in lipid membranes. Nat. Commun. 2024, 15, 3521 10.1038/s41467-024-47803-6. 38664456 PMC11045845

[ref16] Chan D. I.; Prenner E. J.; Vogel H. J. Tryptophan-and arginine-rich antimicrobial peptides: structures and mechanisms of action. Biochim. Biophys. Acta, Biomembr. 2006, 1758, 1184–1202. 10.1016/j.bbamem.2006.04.006. 16756942

[ref17] Afonin S.; Koniev S.; Préau L.; Takamiya M.; Strizhak A. V.; Babii O.; Hrebonkin A.; Pivovarenko V. G.; Dathe M.; le Noble F.; et al. In vivo behavior of the antibacterial peptide cyclo [RRRWFW], explored using a 3-hydroxychromone-derived fluorescent amino acid. Front. Chem. 2021, 9, 688446 10.3389/fchem.2021.688446. 34262894 PMC8273159

[ref18] Rydberg H. A.; Carlsson N.; Nordén B. Membrane interaction and secondary structure of de novo designed arginine-and tryptophan peptides with dual function. Biochem. Biophys. Res. Commun. 2012, 427, 261–265. 10.1016/j.bbrc.2012.09.030. 22989747

[ref19] Rydberg H. A.; Matson M.; Amand H. L.; Esbjörner E. K.; Nordén B. Effects of tryptophan content and backbone spacing on the uptake efficiency of cell-penetrating peptides. Biochemistry 2012, 51, 5531–5539. 10.1021/bi300454k. 22712882

[ref20] Jobin M.-L.; Blanchet M.; Henry S.; Chaignepain S.; Manigand C.; Castano S.; Lecomte S.; Burlina F.; Sagan S.; Alves I. D. The role of tryptophans on the cellular uptake and membrane interaction of arginine-rich cell penetrating peptides. Biochim. Biophys. Acta 2015, 1848, 593–602. 10.1016/j.bbamem.2014.11.013. 25445669

[ref21] Edwards I. A.; Elliott A. G.; Kavanagh A. M.; Zuegg J.; Blaskovich M. A. T.; Cooper M. A. Contribution of Amphipathicity and Hydrophobicity to the Antimicrobial Activity and Cytotoxicity of beta-Hairpin Peptides. ACS Infect. Dis. 2016, 2, 442–450. 10.1021/acsinfecdis.6b00045. 27331141 PMC4906375

[ref22] Hollmann A.; Martínez M.; Noguera M. E.; Augusto M. T.; Disalvo A.; Santos N. C.; Semorile L.; Maffía P. C. Role of amphipathicity and hydrophobicity in the balance between hemolysis and peptide-membrane interactions of three related antimicrobial peptides. Colloids Surf., B 2016, 141, 528–536. 10.1016/j.colsurfb.2016.02.003. 26896660

[ref23] Takahashi H.; Caputo G. A.; Vemparala S.; Kuroda K. Synthetic Random Copolymers as a Molecular Platform To Mimic Host-Defense Antimicrobial Peptides. Bioconjugate Chem. 2017, 28, 1340–1350. 10.1021/acs.bioconjchem.7b00114. 28379682

[ref24] Ergene C.; Yasuhara K.; Palermo E. F. Biomimetic antimicrobial polymers: recent advances in molecular design. Polym. Chem. 2018, 9, 2407–2427. 10.1039/C8PY00012C.

[ref25] Shi S.; Markl A. M.; Lu Z.; Liu R.; Hoernke M. Interplay of Fusion, Leakage, and Electrostatic Lipid Clustering: Membrane Perturbations by a Hydrophobic Antimicrobial Polycation. Langmuir 2022, 38, 2379–2391. 10.1021/acs.langmuir.1c03445. 35148117

[ref26] Breunig M.; Lungwitz U.; Liebl R.; Goepferich A. Breaking up the correlation between efficacy and toxicity for nonviral gene delivery. Proc. Natl. Acad. Sci. U.S.A. 2007, 104, 14454–14459. 10.1073/pnas.0703882104. 17726101 PMC1964826

[ref27] Zhang Y.; Satterlee A.; Huang L. In Vivo Gene Delivery by Nonviral Vectors: Overcoming Hurdles?. Mol. Ther. 2012, 20, 1298–1304. 10.1038/mt.2012.79. 22525514 PMC3392980

[ref28] Richter F.; Leer K.; Martin L.; Mapfumo P.; Solomun J. I.; Kuchenbrod M. T.; Hoeppener S.; Brendel J. C.; Traeger A. The impact of anionic polymers on gene delivery: how composition and assembly help evading the toxicity-efficiency dilemma. J. Nanobiotechnol. 2021, 19, 292 10.1186/s12951-021-00994-2. PMC8477462 34579715

[ref29] Junkes C.; Wessolowski A.; Farnaud S.; Evans R. W.; Good L.; Bienert M.; Dathe M. The interaction of arginine-and tryptophan-rich cyclic hexapeptides with Escherichia coli membranes. J. Pept. Sci. 2008, 14, 535–543. 10.1002/psc.940. 17985396

[ref30] Strom M. B.; Haug B. E.; Skar M. L.; Stensen W.; Stiberg T.; Svendsen J. S. The Pharmacophore of Short Cationic Antibacterial Peptides. J. Med. Chem. 2003, 46, 1567–1570. 10.1021/jm0340039. 12699374

[ref31] Dathe M.; Nikolenko H.; Klose J.; Bienert M. Cyclization increases the antimicrobial activity and selectivity of arginine-and tryptophan-containing hexapeptides. Biochemistry 2004, 43, 9140–9150. 10.1021/bi035948v. 15248771

[ref32] Oh D.; Sun J.; Nasrolahi Shirazi A.; LaPlante K. L.; Rowley D. C.; Parang K. Antibacterial Activities of Amphiphilic Cyclic Cell-Penetrating Peptides against Multidrug-Resistant Pathogens. Mol. Pharmaceutics 2014, 11, 3528–3536. 10.1021/mp5003027. PMC4186684 25157458

[ref33] Junkes C.; Harvey R. D.; Bruce K. D.; Dölling R.; Bagheri M.; Dathe M. Cyclic antimicrobial R-, W-rich peptides: the role of peptide structure and E. coli outer and inner membranes in activity and the mode of action. Eur. Biophys. J. 2011, 40, 515–528. 10.1007/s00249-011-0671-x. 21286704

[ref34] Scheinpflug K.; Nikolenko H.; Komarov I. V.; Rautenbach M.; Dathe M. What goes around comes around-a comparative study of the influence of chemical modifications on the antimicrobial properties of small cyclic peptides. Pharmaceuticals 2013, 6, 1130–1144. 10.3390/ph6091130. 24276422 PMC3818835

[ref35] Bagheri M.; Keller S.; Dathe M. Interaction of W-substituted analogs of cyclo-RRRWFW with bacterial lipopolysaccharides: the role of the aromatic cluster in antimicrobial activity. Antimicrob. Agents Chemother. 2011, 55, 788–797. 10.1128/AAC.01098-10. 21098244 PMC3028809

[ref36] Scheinpflug K.; Krylova O.; Nikolenko H.; Thurm C.; Dathe M. Evidence for a novel mechanism of antimicrobial action of a cyclic R-, W-rich hexapeptide. PLoS One 2015, 10, e0125056 10.1371/journal.pone.0125056. 25875357 PMC4398456

[ref37] Scheinpflug K.; Wenzel M.; Krylova O.; Bandow J. E.; Dathe M.; Strahl H. Antimicrobial peptide cWFW kills by combining lipid phase separation with autolysis. Sci. Rep. 2017, 7, 44332 10.1038/srep44332. 28276520 PMC5343580

[ref38] Wenzel M.; Chiriac A. I.; Otto A.; Zweytick D.; May C.; Schumacher C.; Gust R.; Albada H. B.; Penkova M.; Krämer U.; et al. Small cationic antimicrobial peptides delocalize peripheral membrane proteins. Proc. Natl. Acad. Sci. U.S.A. 2014, 111, E1409–E1418. 10.1073/pnas.1319900111. 24706874 PMC3986158

[ref39] Arouri A.; Dathe M.; Blume A. Peptide induced demixing in PG/PE lipid mixtures: a mechanism for the specificity of antimicrobial peptides towards bacterial membranes?. Biochim. Biophys. Acta, Biomembr. 2009, 1788, 650–659. 10.1016/j.bbamem.2008.11.022. 19118516

[ref40] Finger S.; Kerth A.; Dathe M.; Blume A. The efficacy of trivalent cyclic hexapeptides to induce lipid clustering in PG/PE membranes correlates with their antimicrobial activity. Biochim. Biophys. Acta, Biomembr. 2015, 1848, 2998–3006. 10.1016/j.bbamem.2015.09.012. 26367060

[ref41] Finger S.; Kerth A. M.; Dathe M.; Blume A. The impact of non-ideality of lipid mixing on peptide induced lipid clustering. Biochim. Biophys. Acta, Biomembr. 2020, 1862, 183248 10.1016/j.bbamem.2020.183248. 32145281

[ref42] Liu Z.; Brady A.; Young A.; Rasimick B.; Chen K.; Zhou C.; Kallenbach N. R. Length effects in antimicrobial peptides of the (RW)n series. Antimicrob. Agents Chemother. 2007, 51, 597–603. 10.1128/AAC.00828-06. 17145799 PMC1797765

[ref43] Haug B. E.; Stensen W.; Kalaaji M.; Rekdal O.; Svendsen J. S. Synthetic Antimicrobial Peptidomimetics with Therapeutic Potential. J. Med. Chem. 2008, 51, 4306–4314. 10.1021/jm701600a. 18570363

[ref44] Clark S.; Jowitt T. A.; Harris L. K.; Knight C. G.; Dobson C. B. The lexicon of antimicrobial peptides: a complete set of arginine and tryptophan sequences. Commun. Biol. 2021, 4, 605 10.1038/s42003-021-02137-7. 34021253 PMC8140080

[ref45] Appelt C.; Wessolowski A.; Dathe M.; Schmieder P. Structures of cyclic, antimicrobial peptides in a membrane-mimicking environment define requirements for activity. J. Pept. Sci. 2008, 14, 524–527. 10.1002/psc.924. 17985394

[ref46] Beck K.; Nandy J.; Hoernke M. Membrane permeabilization can be crucially biased by a fusogenic lipid composition−leaky fusion caused by antimicrobial peptides in model membranes. Soft Matter 2023, 19, 2919–2931. 10.1039/D2SM01691E. 37010846

[ref47] Epand R. F.; Savage P. B.; Epand R. M. Bacterial lipid composition and the antimicrobial efficacy of cationic steroid compounds (Ceragenins). Biochim. Biophys. Acta, Biomembr. 2007, 1768, 2500–2509. 10.1016/j.bbamem.2007.05.023. 17599802

[ref48] Ramos-Martín F.; D’Amelio N. Biomembrane lipids: When physics and chemistry join to shape biological activity. Biochimie 2022, 203, 118–138. 10.1016/j.biochi.2022.07.011. 35926681

[ref49] Bartlett G. R. Phosphorus assay in column chromatography. J. Biol. Chem. 1959, 234, 466–468. 10.1016/S0021-9258(18)70226-3. 13641241

[ref50] Parasassi T.; De Stasio G.; Ravagnan G.; Rusch R.; Gratton E. Quantitation of lipid phases in phospholipid vesicles by the generalized polarization of Laurdan fluorescence. Biophys. J. 1991, 60, 179–189. 10.1016/S0006-3495(91)82041-0. 1883937 PMC1260049

[ref51] Harris F. M.; Best K. B.; Bell J. D. Use of laurdan fluorescence intensity and polarization to distinguish between changes in membrane fluidity and phospholipid order. Biochim. Biophys. Acta, Biomembr. 2002, 1565, 123–128. 10.1016/S0005-2736(02)00514-X. 12225860

[ref52] Färber N.; Westerhausen C. Broad lipid phase transitions in mammalian cell membranes measured by Laurdan fluorescence spectroscopy. Biochim. Biophys. Acta, Biomembr. 2022, 1864, 183794 10.1016/j.bbamem.2021.183794. 34627747

[ref53] Struck D. K.; Hoekstra D.; Pagano R. E. Use of resonance energy transfer to monitor membrane fusion. Biochemistry 1981, 20, 4093–4099. 10.1021/bi00517a023. 7284312

[ref54] Shi S.; Fan H.; Hoernke M. Leaky membrane fusion: an ambivalent effect induced by antimicrobial polycations. Nanoscale Adv. 2022, 4, 5109–5122. 10.1039/D2NA00464J. 36504745 PMC9680940

[ref55] Patel H.; Tscheka C.; Heerklotz H. Characterizing vesicle leakage by fluorescence lifetime measurements. Soft Matter 2009, 5, 2849–2851. 10.1039/b908524f.

[ref56] Hadicke A.; Blume A. Binding of short cationic peptides (KX) 4K to negatively charged DPPG monolayers: competition between electrostatic and hydrophobic interactions. Langmuir 2015, 31, 12203–12214. 10.1021/acs.langmuir.5b02882. 26479457

[ref57] Blume A. A comparative study of the phase transitions of phospholipid bilayers and monolayers. Biochim. Biophys. Acta, Biomembr. 1979, 557, 32–44. 10.1016/0005-2736(79)90087-7. 549642

[ref58] Hoernke M.; Schwieger C.; Kerth A.; Blume A. Binding of cationic pentapeptides with modified side chain lengths to negatively charged lipid membranes: Complex interplay of electrostatic and hydrophobic interactions. Biochim. Biophys. Acta, Biomembr. 2012, 1818, 1663–1672. 10.1016/j.bbamem.2012.03.001. 22433675

[ref59] Seelig J. Thermodynamics of lipid-peptide interactions. Biochim. Biophys. Acta, Biomembr. 2004, 1666, 40–50. 10.1016/j.bbamem.2004.08.004. 15519307

[ref60] Mertins O.; Dimova R. Binding of chitosan to phospholipid vesicles studied with isothermal titration calorimetry. Langmuir 2011, 27, 5506–5515. 10.1021/la200553t. 21466162

[ref61] Roversi D.; Troiano C.; Salnikov E.; Giordano L.; Riccitelli F.; De Zotti M.; Casciaro B.; Loffredo M. R.; Park Y.; Formaggio F.; et al. Effects of antimicrobial peptides on membrane dynamics: A comparison of fluorescence and NMR experiments. Biophys. Chem. 2023, 300, 107060 10.1016/j.bpc.2023.107060. 37336097

[ref62] Jurkiewicz P.; Olżyńska A.; Langner M.; Hof M. Headgroup hydration and mobility of DOTAP/DOPC bilayers: a fluorescence solvent relaxation study. Langmuir 2006, 22, 8741–8749. 10.1021/la061597k. 17014112

[ref63] Cametti C. Polyion-induced aggregation of oppositely charged liposomes and charged colloidal particles: The many facets of complex formation in low-density colloidal systems. Chem. Phys. Lipids 2008, 155, 63–73. 10.1016/j.chemphyslip.2008.07.009. 18718458

[ref64] Heerklotz H. Membrane stress and permeabilization induced by asymmetric incorporation of compounds. Biophys. J. 2001, 81, 184–195. 10.1016/S0006-3495(01)75690-1. 11423405 PMC1301502

[ref65] Wimley W. C.; Hristova K. The mechanism of membrane permeabilization by peptides: still an enigma. Aust. J. Chem. 2020, 73, 96–103. 10.1071/CH19449. PMC7185740 32341596

[ref66] Shi S.; Quarta N.; Zhang H.; Lu Z.; Hof M.; Šachl R.; Liu R.; Hoernke M. Hidden complexity in membrane permeabilization behavior of antimicrobial polycations. Phys. Chem. Chem. Phys. 2021, 23, 1475–1488. 10.1039/D0CP05651K. 33399594

[ref67] Bortolotti A.; Troiano C.; Bobone S.; Konai M.; Ghosh C.; Bocchinfuso G.; Acharya Y.; Santucci V.; Bonacorsi S.; Di Stefano C.; Haldar J.; Stella L. Mechanism of lipid bilayer perturbation by bactericidal membrane-active small molecules. Biochim. Biophys. Acta, Biomembr. 2023, 1865, 184079 10.1016/j.bbamem.2022.184079. 36374761

[ref68] Steigenberger J.; Verleysen Y.; Geudens N.; Madder A.; Martins J. C.; Heerklotz H. Complex electrostatic effects on the selectivity of membrane-permeabilizing cyclic lipopeptides. Biophys. J. 2023, 122, 950–963. 10.1016/j.bpj.2022.07.033. 35927958 PMC10111218

[ref69] Domingues T. M.; Perez K. R.; Riske K. A. Revealing the Mode of Action of Halictine Antimicrobial Peptides: A Comprehensive Study with Model Membranes. Langmuir 2020, 36, 5145–5155. 10.1021/acs.langmuir.0c00282. 32336099

[ref70] Poojari C. S.; Scherer K. C.; Hub J. S. Free energies of membrane stalk formation from a lipidomics perspective. Nat. Commun. 2021, 12, 6594 10.1038/s41467-021-26924-2. 34782611 PMC8593120

[ref71] Wimley W. C. Describing the mechanism of antimicrobial peptide action with the interfacial activity model. ACS Chem. Biol. 2010, 5, 905–917. 10.1021/cb1001558. 20698568 PMC2955829

[ref72] Al Nahas K.; Fletcher M.; Hammond K.; Nehls C.; Cama J.; Ryadnov M. G.; Keyser U. F. Measuring Thousands of Single-Vesicle Leakage Events Reveals the Mode of Action of Antimicrobial Peptides. Anal. Chem. 2022, 94, 9530–9539. 10.1021/acs.analchem.1c03564. 35760038 PMC9280716

[ref73] Krauson A. J.; He J.; Wimley A. W.; Hoffmann A. R.; Wimley W. C. Synthetic Molecular Evolution of Pore-Forming Peptides by Iterative Combinatorial Library Screening. ACS Chem. Biol. 2013, 8, 823–831. 10.1021/cb300598k. 23394375 PMC3644507

[ref74] Pourmasoumi F.; Hengoju S.; Beck K.; Stephan P.; Klopfleisch L.; Hoernke M.; Rosenbaum M. A.; Kries H. Analysing Megasynthetase Mutants at High Throughput Using Droplet Microfluidics. ChemBioChem 2023, 24, e202300680 10.1002/cbic.202300680. 37804133

[ref75] Van Oort C. M.; Ferrell J. B.; Remington J. M.; Wshah S.; Li J. AMPGAN v2: Machine Learning-Guided Design of Antimicrobial Peptides. J. Chem. Inf. Model. 2021, 61, 2198–2207. 10.1021/acs.jcim.0c01441. 33787250 PMC8281497

[ref76] Yan J.; Cai J.; Zhang B.; Wang Y.; Wong D. F.; Siu S. W. Recent progress in the discovery and design of antimicrobial peptides using traditional machine learning and deep learning. Antibiotics 2022, 11, 1451 10.3390/antibiotics11101451. 36290108 PMC9598685

