# Supplementary material for: Strong Membrane Permeabilization Activity Can Reduce Selectivity of Cyclic Antimicrobial Peptides
Source: J Phys Chem B. 2025 Feb 19;129(9):2446–60. doi: 10.1021/acs.jpcb.4c05019 (PMC11891913; doi:10.1021/acs.jpcb.4c05019)
Supplement: Supplementary file 1 — jp4c05019_si_001.pdf [file jp4c05019_si_001.pdf]

Supporting Information

Strong Membrane Permeabilization Activity Can Reduce Selectivity of Cyclic Antimicrobial Peptides

Katharina Beck[1,2,3], Janina Nandy[1,4], and Maria Hoernke[1,5]

[1] *Pharmaceutical Technology and Biopharmacy, Institute of Pharmaceutical Sciences, University of Freiburg, Germany*
[2] *Physiology, Institute of Theoretical Medicine, University of Augsburg, Germany*
[3] *Experimental Physics I, Institute of Physics, University of Augsburg, Germany*
[4] *currently: Division of Biophysics, Research Center Borstel, Leibniz Lung Center, Germany*
[5] *Physical Chemistry, Martin-Luther-Universität Halle (S.), Germany*

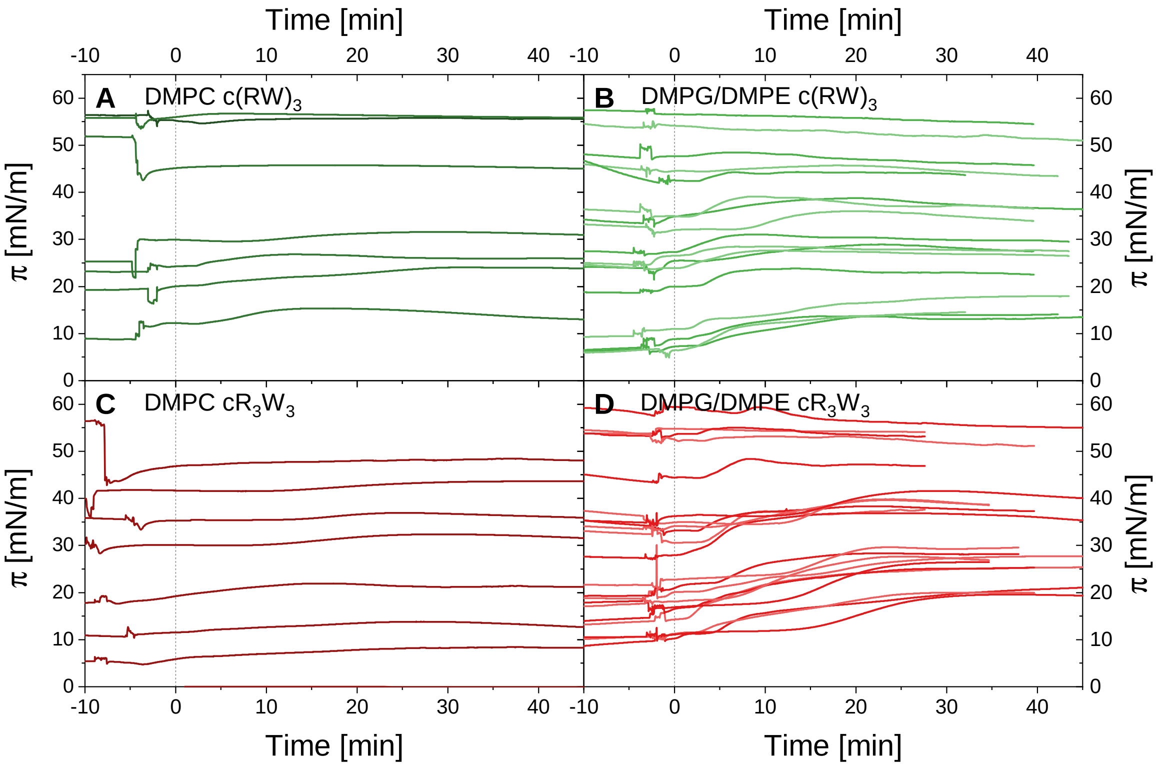

Figure SI.1: The surface pressure π of lipid monolayers with various initial pressures π_0 after the addition of antimicrobial peptides to the subphase (final concentration 900 nM) is shown as a function of time. The resulting changes in surface pressure, $\Delta\pi$, observed at equilibrium (after approximately 35 minutes), are presented in Figure 1. (A) $c(RW)_3$ adsorption to DMPC monolayers, (B) $c(RW)_3$ adsorption to DMPG/DMPE (1:1) monolayers, (C) cR_3W_3 adsorption to DMPC monolayers, and (D) cR_3W_3 adsorption to DMPG/DMPE (1:1) monolayers. (10 mM TRIS; 110 mM NaCl; 0.5 mM EDTA; pH 7.4; 20,°C)

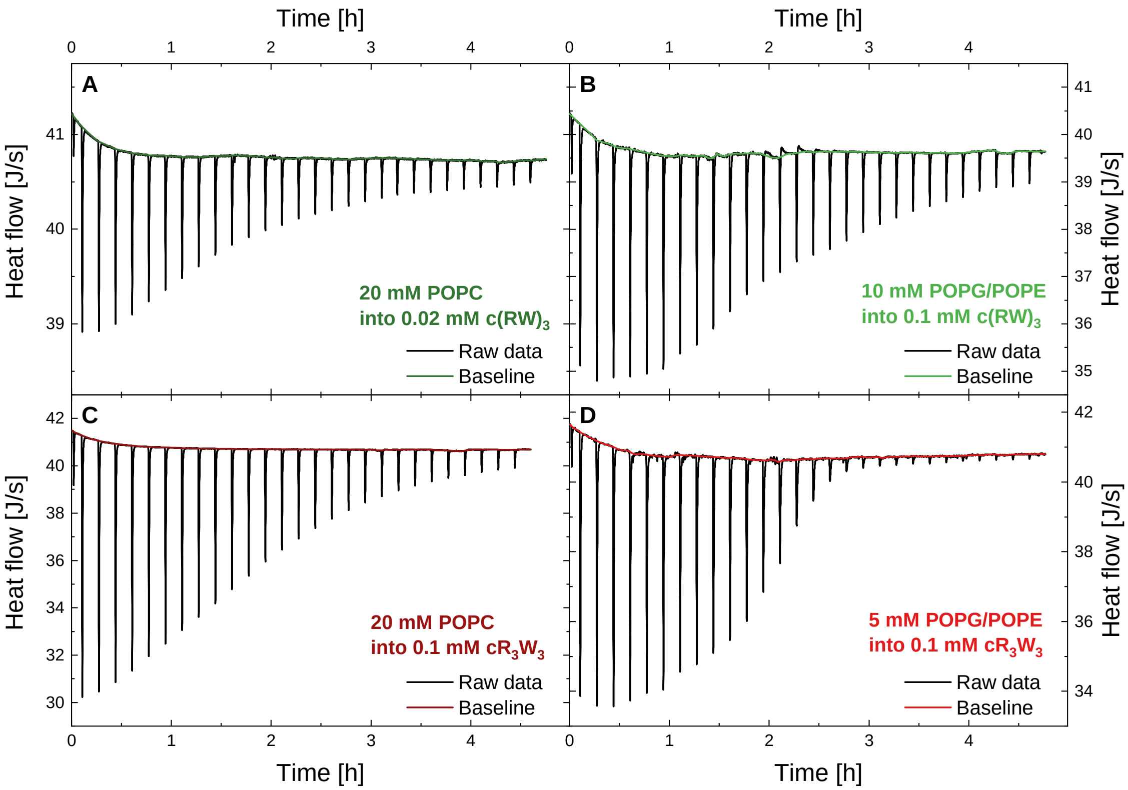

Figure SI.2: ITC raw thermograms obtained from the titration of liposome suspension in steps of 10 µL injected every 10 minutes into aqueous solutions of antimicrobial peptides. (A) 20 mM POPC liposome suspension was titrated into 0.02 mM c(RW)$_3$ solution, (B) 10 mM POPG/POPE (1:1) liposome suspension was titrated into 0.1 mM c(RW)$_3$ solution, (C) 20 mM POPC liposome suspension was titrated into 0.1 mM cR$_3$W$_3$ solution, and (D) 5 mM POPG/POPE (1:1) liposome suspension was titrated into 0.1 mM cR$_3$W$_3$ solution. The coloured lines represent the baseline used for calculating the integrated heat per injection, shown in Figure 2. (10 mM TRIS; 110 mM NaCl; 0.5 mM EDTA; pH 7.4; 25 °C)

ITC data was fitted using the built-in "Single Set of Identical Sites" model in the MicroCal Origin Analysis Software (MicroCal, Northhampton, MA, USA) based on Origin 7. This simplified model assumes a single type of binding interaction between the peptide and the lipid molecules and combines all electrostatic and hydrophobic contributions to the binding. The binding isotherm is described by the following equation:

$$Q = \frac{n \cdot M_t \cdot \Delta H \cdot V_0}{2} \left[1 + \frac{X_t}{nM_t} + \frac{1}{nKM_t} - \sqrt{\left(1 + \frac{X_t}{nM_t} + \frac{1}{nKM_t}\right)^2 - \frac{4X_t}{nM_t}} \right] \tag{8}$$

where Q is the total heat released, n is the number of binding sites, K is the binding constant, ΔH is the binding enthalpy, M_t is the bulk concentration of peptides, X_t is the bulk concentration of lipids, and V_0 is the volume of the ITC cell. Including the site-size parameter n allows the model to account for varying stoichiometry in the binding process.

This simplified model can be more appropriate for elucidating fundamental differences in binding behaviour, particularly when the assumptions required for more complex approaches are not adequately met. The antimicrobial membrane-active peptides used in this study affects the charge distribution on and within lipid membranes. This deviation from the uniform charge distribution assumed by models such as the Gouy-Chapman model significantly compromises their applicability and interpretability in our system,[59] rendering more complex approaches less advantageous compared to the simpler model we used.

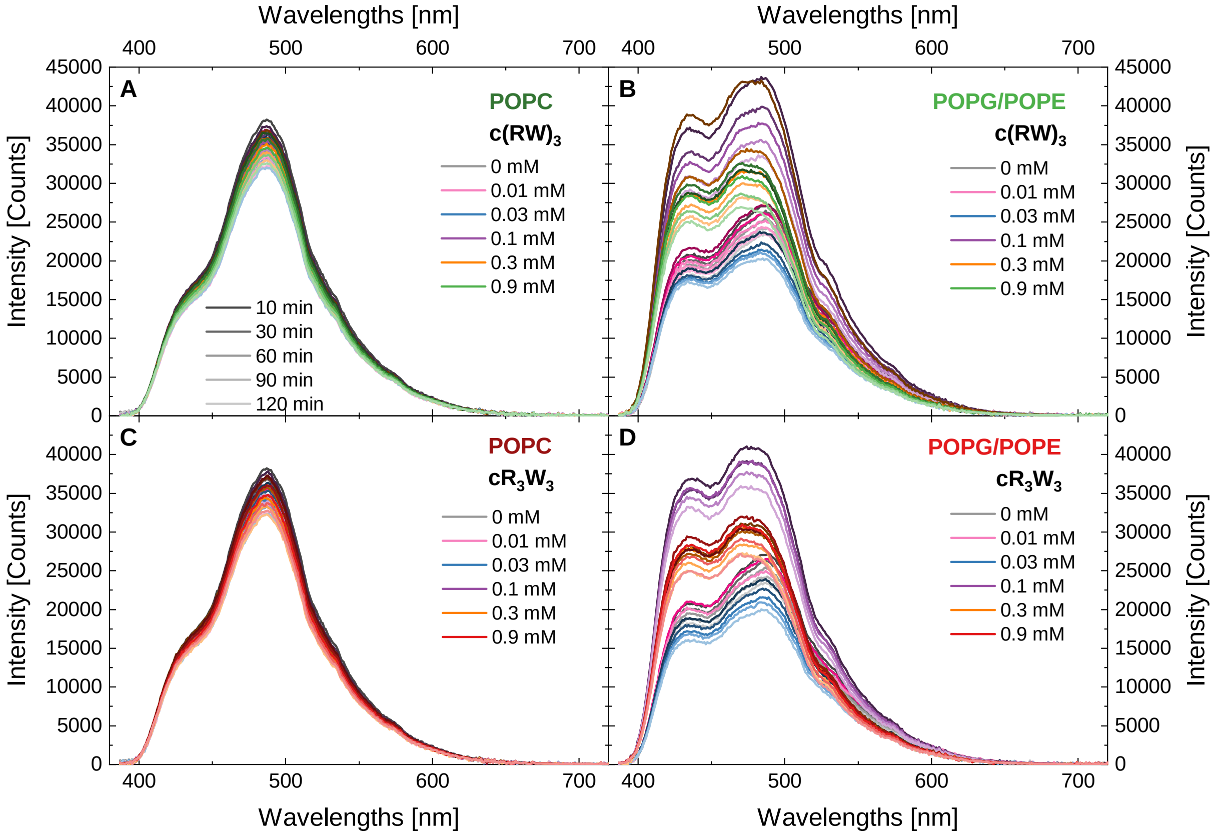

Figure SI.3: Emission spectra obtained from fluorescence spectroscopy of membrane-embedded laurdan. Liposome suspensions (0.3 mM lipids) were incubated with antimicrobial peptides for up to 2 hours. c(RW)$_3$ was added to (A) POPC and (B) POPG/POPE (1:1) liposomes. cR$_3$W$_3$ was added to (C) POPC and (D) POPG/POPE (1:1) liposomes. Colours indicate peptide concentrations, while colour intensities represent different incubation times. A shift in emission intensity maxima reflects changes in lipid headgroup packing and membrane fluidity. Figure 3 presents these membrane alterations as ΔGP. (10 mM TRIS; 110 mM NaCl; 0.5 mM EDTA; pH 7.4; 25 °C)

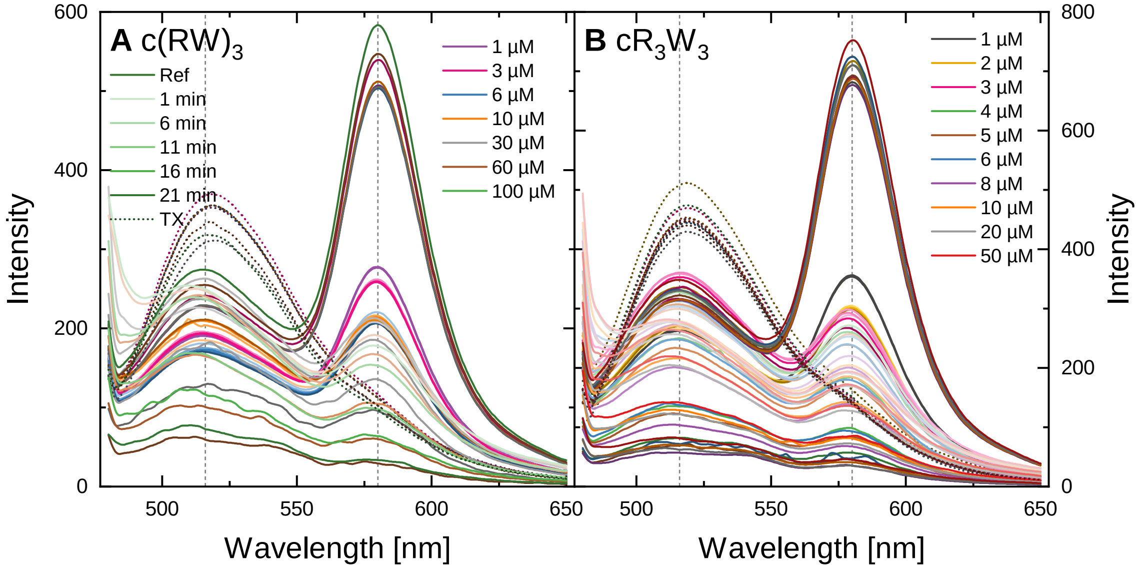

Figure SI.4: Emission spectra obtained from fluorescence spectroscopy of liposomes containing NBD- and Rhodamine-labeled lipids incubated with unlabeled liposomes (1:4) and peptides. 30 µM POPG/POPE (1:1) liposomes were incubated with various concentrations of (A) c(RW)$_3$ and (B) cR$_3$W$_3$. Colours indicate peptide concentrations, while colour intensities represent different incubation times. For the calculation of Lipid Mixing Efficiency LME shown in Figure 5, spectra were also recorded before peptide addition (Ref) and after addition of Triton X-100 (TX). Data showing a decrease in NBD intensity exceeding 50 % are depicted in grey in Figure 5. (10 mM TRIS; 110 mM NaCl; 0.5 mM EDTA; pH 7.4; 25 °C)

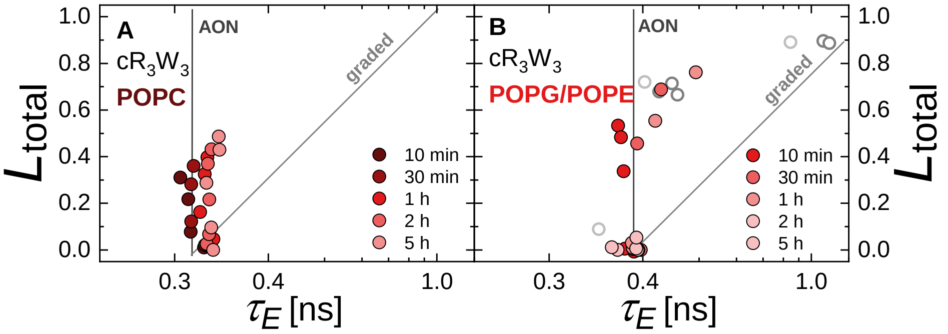

Figure SI.5: Calcein leakage reveals details of the permeabilization behaviour of cR$_3$W$_3$, in particular all-or-none leakage behaviour. Supplementary to Figure 6, the total vesicle leakage L_{total} of 30 µM liposome suspensions is shown as a function of the fluorescence lifetime of entrapped calcein, τ_F, on a reciprocal scale. (A) POPC liposomes and (B) POPG/POPE (1:1) liposomes were incubated with cR$_3$W$_3$. The theoretical behaviour of AON (all-or-none) and graded leakage is shown as grey lines. As illustrated in Figure SI.6, data with a decrease in *Sum of B* of more than 20% is depicted in grey. (10 mM TRIS; 110 mM NaCl; 0.5 mM EDTA; pH 7.4; 25 °C)

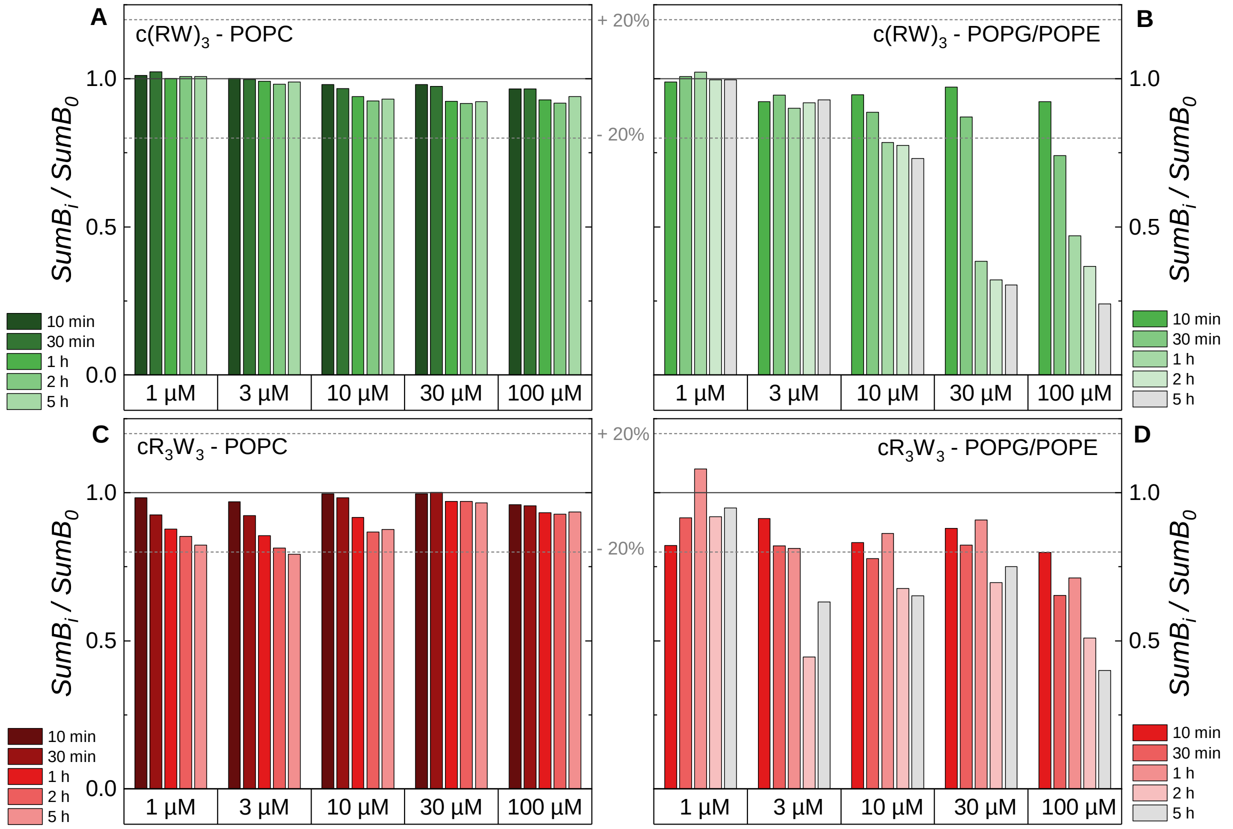

Figure SI.6: The *Sum of B* values corresponding to the calcein leakage data presented in Figure 6. Relative changes in *Sum of B* for 30 µM liposome suspensions are shown as a function of peptide concentration with increasing incubation time. (A) POPC liposomes were incubated with c(RW)$_3$, (B) POPG/POPE (1:1) liposomes were incubated with c(RW)$_3$, (C) POPC liposomes were incubated with cR$_3$W$_3$, and (D) POPG/POPE (1:1) liposomes were incubated with cR$_3$W$_3$. The 20 % deviation threshold is indicated, and data points beyond this threshold are depicted in grey in Figure 6 and Figure SI.5. (10 mM TRIS; 110 mM NaCl; 0.5 mM EDTA; pH 7.4; 25 °C)

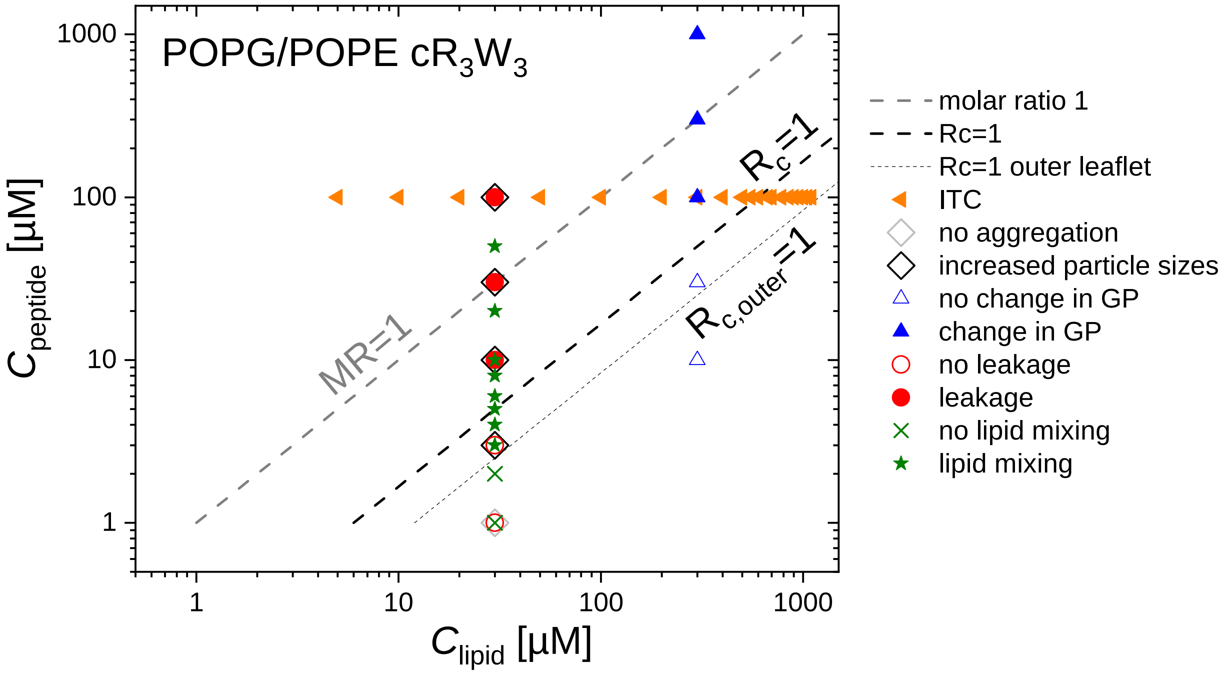

Figure SI.7: Comparison of experimental conditions, *i.e.* peptide and lipid concentrations, for various methods presented in the paper. The onset of vesicle aggregation, lipid mixing, changes in laurdan generalized polarization, and leakage coincide with the neutralization of the lipid and peptides charges ($R_c = 1$).

Table SI.1: Summary of the amount of entrapped calcein B_E with the corresponding fluorescence lifetime τ_E and the amount of free calcein B_F with τ_F, as derived from the biexponential fit of the acquired fluorescence decay curves corresponding to data in Figure 6A. Calcein-filled POPC liposomes were incubated with c(RW)$_3$.

**POPC - c(RW)$_3$**		B_E	τ_E	B_F	τ_F
0 µM	10 min	34600	0.34	2400	3.24
	30 min	34500	0.34	2400	3.24
	1 h	35100	0.34	2500	3.25
	2 h	35000	0.34	2500	3.27
	5 h	34900	0.34	2600	3.28
1 µM	10 min	35000	0.34	2400	3.25
	30 min	35300	0.34	2400	3.25
	1 h	35100	0.34	2500	3.27
	2 h	35200	0.34	2600	3.27
	5 h	35200	0.34	2600	3.31
3 µM	10 min	34600	0.34	2500	3.25
	30 min	34400	0.34	2500	3.28
	1 h	34700	0.34	2600	3.31
	2 h	34300	0.34	2600	3.31
	5 h	34400	0.34	2800	3.37
10 µM	10 min	33900	0.34	2500	3.30
	30 min	33300	0.34	2600	3.35
	1 h	32800	0.34	2700	3.38
	2 h	32100	0.34	2800	3.41
	5 h	32100	0.34	3100	3.48
30 µM	10 min	33800	0.33	2600	3.32
	30 min	33300	0.33	2800	3.39
	1 h	32000	0.34	3000	3.47
	2 h	31500	0.34	3200	3.51
	5 h	31300	0.34	3700	3.56
100 µM	10 min	33100	0.33	2800	3.41
	30 min	32600	0.33	3300	3.49
	1 h	31500	0.34	3700	3.58
	2 h	30500	0.34	4400	3.64
	5 h	30300	0.36	5500	3.70

Table SI.2: Summary of the amount of entrapped calcein B_E with the corresponding fluorescence lifetime τ_E and the amount of free calcein B_F with τ_F, as derived from the biexponential fit of the acquired fluorescence decay curves corresponding to data in Figure 6B. Calcein-filled POPG/POPE (1:1) liposomes were incubated with c(RW)$_3$.

**POPG/POPE - c(RW)$_3$**		B_E	τ_E	B_F	τ_F
0 µM	10 min	20300	0.39	2200	3.33
	30 min	20800	0.39	2200	3.34
	1 h	21300	0.39	2210	3.33
	2 h	21000	0.39	2270	3.31
	5 h	20700	0.38	2230	3.32
1 µM	10 min	20100	0.37	2220	3.36
	30 min	20900	0.37	2320	3.38
	1 h	21600	0.37	2370	3.37
	2 h	20900	0.37	2390	3.39
	5 h	20500	0.37	2350	3.41
3 µM	10 min	18800	0.37	2100	3.36
	30 min	19700	0.36	2230	3.40
	1 h	19100	0.37	2360	3.43
	2 h	19200	0.37	2420	3.45
	5 h	19000	0.36	2460	3.49
10 µM	10 min	19200	0.36	2300	3.43
	30 min	18200	0.35	2500	3.51
	1 h	16200	0.36	2830	3.56
	2 h	15600	0.35	3050	3.64
	5 h	14300	0.35	3250	3.69
30 µM	10 min	19900	0.35	2060	3.36
	30 min	18000	0.35	2360	3.50
	1 h	7900	0.34	2530	3.73
	2 h	6000	0.36	3160	3.81
	5 h	5000	0.34	3820	3.85
100 µM	10 min	18400	0.33	2610	3.56
	30 min	14500	0.33	3360	3.71
	1 h	8400	0.33	4190	3.81
	2 h	5500	0.33	4930	3.86
	5 h	1900	0.41	5890	3.92

Table SI.3: Summary of the amount of entrapped calcein B_E with the corresponding fluorescence lifetime τ_E and the amount of free calcein B_F with τ_F, as derived from the biexponential fit of the acquired fluorescence decay curves corresponding to data in Figure 6C. Calcein-filled POPC liposomes were incubated with cR_3W_3.

**POPC - cR_3W_3**		B_E	τ_E	B_F	τ_F
0 µM	10 min	34800	0.33	2230	3.21
	30 min	34800	0.33	2320	3.23
	1 h	34600	0.33	2380	3.23
	2 h	34800	0.33	2450	3.26
	5 h	35300	0.33	2580	3.29
1 µM	10 min	33900	0.33	2630	3.30
	30 min	31700	0.33	2800	3.40
	1 h	29800	0.33	3040	3.44
	2 h	28900	0.33	3350	3.51
	5 h	28100	0.33	3740	3.58
3 µM	10 min	33100	0.33	3060	3.40
	30 min	31000	0.33	3600	3.50
	1 h	28200	0.33	4020	3.59
	2 h	26300	0.33	4730	3.66
	5 h	25300	0.33	5800	3.72
10 µM	10 min	32000	0.31	5460	3.67
	30 min	30000	0.31	7310	3.76
	1 h	26600	0.32	8540	3.80
	2 h	23600	0.33	10260	3.83
	5 h	22000	0.33	13250	3.87
30 µM	10 min	27200	0.31	11300	3.84
	30 min	25000	0.31	14070	3.87
	1 h	22700	0.33	15480	3.88
	2 h	21300	0.33	17410	3.90
	5 h	19500	0.34	20140	3.91
100 µM	10 min	23100	0.30	14670	3.87
	30 min	21300	0.32	16680	3.89
	1 h	19500	0.33	17800	3.90
	2 h	18300	0.33	19120	3.91
	5 h	17000	0.34	21850	3.92

Table SI.4: Summary of the amount of entrapped calcein B_E with the corresponding fluorescence lifetime τ_E and the amount of free calcein B_F with τ_F, as derived from the biexponential fit of the acquired fluorescence decay curves corresponding to data in Figure 6D. Calcein-filled POPG/POPE (1:1) liposomes were incubated with cR_3W_3.

**POPG/POPE - cR_3W_3**		B_E	τ_E	B_F	τ_F
0 µM	10 min	18800	0.39	2020	3.27
	30 min	18700	0.40	2000	3.29
	1 h	18700	0.39	2000	3.28
	2 h	19400	0.39	2080	3.27
	5 h	26900	0.37	2230	3.23
1 µM	10 min	15600	0.39	1880	3.31
	30 min	17000	0.39	2050	3.34
	1 h	18100	0.39	2170	3.34
	2 h	17800	0.39	2210	3.38
	5 h	25300	0.36	2570	3.39
3 µM	10 min	17100	0.38	2120	3.33
	30 min	15100	0.39	2250	3.41
	1 h	13100	0.39	2490	3.54
	2 h	8200	0.39	2630	3.69
	5 h	15600	0.35	4040	3.70
10 µM	10 min	10400	0.37	8360	3.85
	30 min	7900	0.39	9940	3.88
	1 h	6500	0.42	11610	3.91
	2 h	4200	0.42	12810	3.93
	5 h	4000	0.40	14660	3.92
30 µM	10 min	7800	0.37	12660	3.90
	30 min	4800	0.43	14670	3.93
	1 h	3700	0.49	16000	3.94
	2 h	1400	1.12	16650	3.97
	5 h	700	0.84	18450	3.97
100 µM	10 min	7200	0.37	10110	3.90
	30 min	4100	0.46	11710	3.93
	1 h	3400	0.45	12160	3.92
	2 h	1100	1.19	12620	3.97
	5 h	-400	0.00	14900	3.94
